# *Nannochloropsis* plastid and mitochondrial phylogenomes reveal organelle diversification mechanism and intragenus phylotyping strategy in microalgae

**DOI:** 10.1186/1471-2164-14-534

**Published:** 2013-08-05

**Authors:** Li Wei, Yi Xin, Dongmei Wang, Xiaoyan Jing, Qian Zhou, Xiaoquan Su, Jing Jia, Kang Ning, Feng Chen, Qiang Hu, Jian Xu

**Affiliations:** 1 BioEnergy Genome Center and Shandong Key Laboratory of Energy Genetics, Qingdao Institute of BioEnergy and Bioprocess Technology, Chinese Academy of Sciences, Qingdao, Shandong 266101, China; 2 University of Chinese Academy of Sciences, Beijing 100049, China; 3 Laboratory for Algae Research and Biotechnology, Department of Applied Biological Sciences, Arizona State University, Mesa, AZ 85212, USA; 4 Institute of Marine and Environmental Technology, University of Maryland Center for Environmental Science, Baltimore, MD 21202, USA

**Keywords:** *Nannochloropsis*, Plastid phylogenomes, Mitochondrial phylogenomes, Intragenus phylotyping strategy

## Abstract

**Background:**

Microalgae are promising feedstock for production of lipids, sugars, bioactive compounds and in particular biofuels, yet development of sensitive and reliable phylotyping strategies for microalgae has been hindered by the paucity of phylogenetically closely-related finished genomes.

**Results:**

Using the oleaginous eustigmatophyte *Nannochloropsis* as a model, we assessed current intragenus phylotyping strategies by producing the complete plastid (pt) and mitochondrial (mt) genomes of seven strains from six *Nannochloropsis* species. Genes on the pt and mt genomes have been highly conserved in content, size and order, strongly negatively selected and evolving at a rate 33% and 66% of nuclear genomes respectively. Pt genome diversification was driven by asymmetric evolution of two inverted repeats (IRa and IRb): *psbV* and *clpC* in IRb are highly conserved whereas their counterparts in IRa exhibit three lineage-associated types of structural polymorphism via duplication or disruption of whole or partial genes. In the mt genomes, however, a single evolution hotspot varies in copy-number of a 3.5 Kb-long, *cox1*-harboring repeat. The organelle markers (e.g., *cox1*, *cox2*, *psbA*, *rbcL* and *rrn16_mt*) and nuclear markers (e.g., *ITS2* and *18S*) that are widely used for phylogenetic analysis obtained a divergent phylogeny for the seven strains, largely due to low SNP density. A new strategy for intragenus phylotyping of microalgae was thus proposed that includes *(i)* twelve sequence markers that are of higher sensitivity than *ITS2* for interspecies phylogenetic analysis, *(ii)* multi-locus sequence typing based on *rps11_mt*-*nad4*, *rps3_mt* and *cox2*-*rrn16_mt* for intraspecies phylogenetic reconstruction and *(iii)* several SSR loci for identification of strains within a given species.

**Conclusion:**

This first comprehensive dataset of organelle genomes for a microalgal genus enabled exhaustive assessment and searches of all candidate phylogenetic markers on the organelle genomes. A new strategy for intragenus phylotyping of microalgae was proposed which might be generally applicable to other microalgal genera and should serve as a valuable tool in the expanding algal biotechnology industry.

## Background

Microalgae include many evolutionarily diverse lineages of unicellular photosynthetic eukaryotes that range in size from a few to several hundred micrometers. They contribute significantly to the primary production and the biogeochemical cycle of our biosphere [1]. They have also found increasing applications for production of lipids, sugars, bioactive compounds and in particular, biofuels [2].

Cellular functions of present-day microalgae are underpinned by plastid (pt), mitochondrial (mt) and nuclear (nc) genomes. Pt and mt play important roles in the evolution of microalgae and higher plants. The origin of pt has been traced to an endosymbiosis event between eukaryotic cell and cyanobacteria, which occurred around 1.2 Ga ago [3]. The engulfed photosynthetic unicellular cyanobacteria adapted to the environment inside the host cells and eventually became the present day eukaryotic pt [4]. The pt genome, in multiple copies, is inherited in a non-Mendelian fashion. Therefore, the genetic information from pt genome can provide an independent view of the phylogeny of its host organisms. Mt, according to the serial endosymbiosis theory, is the direct descendant of a bacterial endosymbiont (likely an alpha-proteobacterium) that became established in the early evolution of a nucleus-containing (but amitochondriate) host cell [5]. Analysis of microalgal organelle genomes has revealed their endosymbiotic origins [6], frequent gene transfers from organelles to nucleus [7] and the phylogeny among genera [8]. However, evolutionary dynamics of organelle genomes that drive microalgal speciation (i.e., within the genus) remains poorly understood.

Due to their asexual reproduction, slow evolution, few recombination, and relatively simple gene structure and dominance of single-copy genes, organelle genes have often been employed as phylogenetic markers [9], which are essential tools in algal research and biotechnology. Several molecular markers are frequently used for phylotyping algae, including the second internal transcribed spacer (*ITS2*) of nuclear ribosomal DNA (18S rRNA), mitochondrial cytochrome oxidase subunit I (*cox1*), and plastid ribulose-l-5-bisphosphate carboxylase/oxygenase (*rbcL*). However, limitations of the strategy are apparent: (i) different markers frequently gave different phylogenetic scenario (i.e., sub-specificity); (ii) most markers could not distinguish strains within a given species (i.e., sub-sensitivity); (iii) currently available markers could not be applied to microalgae of all kinds (i.e., sub-applicability) [10,11]. For instance, *cox1* is useful mainly for identification of red and brown algae [12-15], whereas *tufA* (encoding plastid elongation factor Tu gene) and *rbcL* serve as the primary DNA barcodes for green algae and diatoms respectively [11,16,17]. However the genomic basis of such practices remains largely unknown. Exhaustive search and comparative assessment of phylogenetic markers have not been possible, largely due to the paucity of complete organelle genomes from phylogenetically closely related strains and species.

*Nannochloropsis* (Eustigmatophyceae) is a genus of unicellular photosynthetic microalgae, ranging in size from 2 to 5 μm and widely distributed in marine, fresh and brackish waters [18-21]. It is an emerging model for photosynthetic production of oil (triacylglycerol; TAG) because of its ability to grow rapidly, synthesize large amounts of TAG and polyunsaturated fatty acids and tolerate a wide range of environmental conditions [22-24]. Traditional approaches for identifying species in *Nannochloropsis* include morphology observation, pigment and fatty acid composition and 18S rRNA sequence analysis [25]. However previous analysis based on *18S* (a nuclear gene) and *rbcL* (a pt gene) resulted in conflicting phylogenies among microalgae lineages that include *Nannochloropsis*[25]. Moreover, the intragenus relationship of *Nannochloropsis* spp. (especially among *N. oculata*, *N. limnetica*, *N. granulata* and *N. oceanica*) was inconsistent among 18S-based phylogenetic trees [20,21]. In this study, using *Nannochloropsis* genus as a model, we assessed current intragenus phylotyping strategies by producing the complete pt and mt genomes of seven strains from six *Nannochloropsis* species. This first comprehensive dataset of organelle genomes for a microalgal genus was employed to dissect the evolutionary dynamics of organelle genomes at the genus, species and strain levels. Furthermore, the dataset enabled exhaustive exploration of novel phylogenetic markers suitable for inter-species and intra-species identification of microalgae. A new strategy for intragenus phylotyping of microalgae was therefore proposed.

## Results and discussion

### Global structural features of the organelle genomes in *Nannochloropsis*

To capture a comprehensive picture of microalgal organelle evolution at the strain-, species- and genus-levels, two *N. oceanica* strains (IMET1 and CCMP531) and one strain from each of other five known species in *Nannochloropsis* Genus: *N. salina* (CCMP537), *N. gaditana* (CCMP527), *N. oculata* (CCMP525), *N. limnetica* (CCMP505) and *N. granulata* (CCMP529) were chosen for sequencing (Methods). The pt and mt genomes of IMET1 were first assembled from whole-genome shotgun reads and then manually finished (Methods). Draft sequences of the other organelle genomes were extracted from whole-genome contigs by BLAST using IMET1 as a reference. Long-range PCR was used to test the orientation of large repeats and bridge the remaining gaps. The four junctions between the inverted repeats and single-copy segments were confirmed by sequencing PCR products. The seven sets of organelle genomes were manually inspected and completely finished (Table 1).

**Table 1:** **Features of the *Nannochloropsis *organelle genomes (Plastid/Mitochondria)**

	***N. oceanica *IMET1**	***N. oceanica *CCMP531**	***N. salina *CCMP537**	***N. gaditana *CCMP527**	***N. oculata *CCMP525**	***N. limnetica *CCMP505**	***N. granulata *CCMP529**
**Size (bp)**	117,548/38,057	117,634/38,057	114,883/41,907	114,867/42,206	117,463/38,444	117,806/38,543	117,672/38,791
**LSC length (bp)**	57,360/-	57,387/-	56,882/-	56,925/-	57,287/-	57,444/-	57,352/-
**SSC length (bp)**	45,235/-	45,240/-	47,364/-	47,698/-	45,227/-	45,259/-	45,247/-
**IR length (bp)**	7,485/-	7,496/-	5,320/-	5,122/-	7,476/-	7,549/-	7,527/-
**Number of genes**	160/63	160/63	156/64	156/64	160/63	160/63	160/63
**Protein-coding genes**	126/35	126/35	123/36	123/36	126/35	126/35	126/35
**Structure RNAs**	34/28	34/28	33/28	33/28	34/28	34/28	34/28
**GC content (%)**	33.6/31.9	33.6/31.9	33.1/31.4	33.0/31.4	33.4/31.8	33.5/31.7	33.4/32.0
**Coding regions (%)**	83.5/87.5	83.4/87.5	83.6/81.4	83.8/80.9	83.5/84.7	83.7/84.6	84.5/84.1

**Figure 1:**
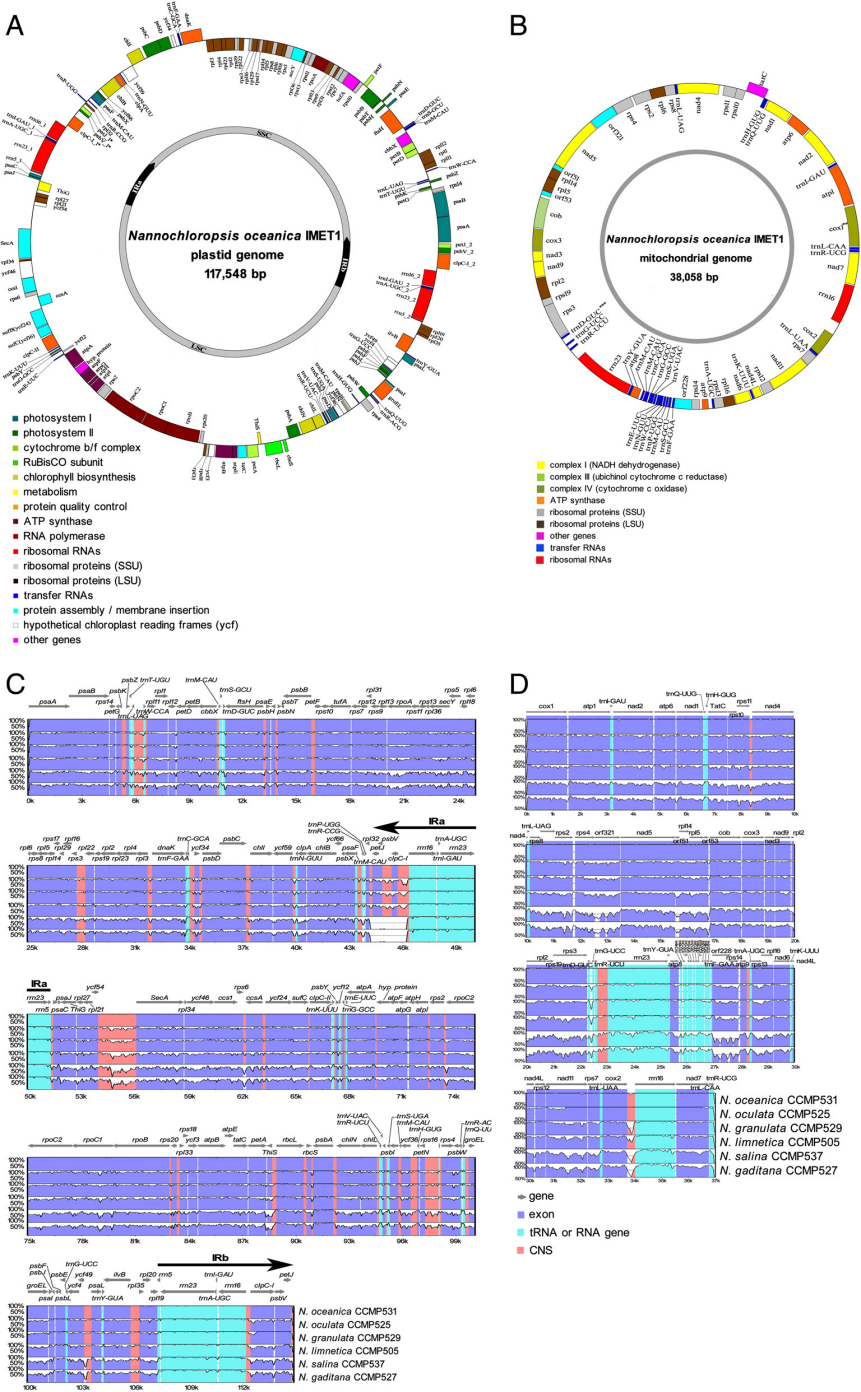
**Plastid and mitochondrial genomes of seven *Nannochloropsis *strains. (A)** Genome map of the complete pt sequence of *N. oceanica* IMET1. **(B)** Genome map of the complete mt sequence of *N. oceanica* IMET1. Genes shown outside the outer circle are transcribed clockwise and those inside are transcribed counter clockwise. Genes belonging to different functional groups are color-coded. Alignment of the *Nannochloropsis* plastid **(C)** and mitochondrial **(D)** genomes were also shown respectively. Genomic regions are color-coded as protein-coding (blue), rRNA/tRNA-coding (cyan) and conserved noncoding sequences (red). * CCMP527 and CCMP537 do not contain the region. **Two copies of *cox1* are present in CCMP527 and CCMP537. ***In CCMP529, *trnD-GUC* was translocated to the interval between *cox2* and *rrn16*.

The circular pt genomes ranged in length from 114,867 to 117,806 bp, with an average GC content of 33.3%. Each pt genome was divided into four structural domains (Figure 1A): a large single copy (LSC), a small single copy (SSC), and inverted repeats (IR) which are present in pinpoint duplicate separated by the two single-copy regions. Such a quadripartite structure was previously found in many other algal pt genomes including the primary endosymbiotic *Chlamydomonas reinhardtii* and secondary endosymbiotic diatoms *Phaeodactylum tricornutum* and *Thalassiosira pseudonana*[26,27].

Each pt genome encodes 152 unique genes including 26 tRNA, three rRNA and 123 proteins. In addition, eight genes (*clpC-I*, *psbV*, *petJ*, *rrn16*, *trnI(gat)*, *trnA(tgc)*, *rrn23* and *rrn5*) were duplicated in the IR of CCMP505, CCMP525, CCMP531, CCMP529 and IMET1, while only five genes (*rrn16*, *trnI(gat)*, *trnA(tgc)*, *rrn23* and *rrn5*) were duplicated in the IR of CCMP527 and CCMP537. The overall genome structure and gene content of *Nannochloropsis* pt are similar to those of *T. pseudonana*, *P. tricornutum* and *Ectocarpus siliculosus*[8,26].

The circular mt genomes were 38,057 ~ 42,206 bp in length (Figure 1B), with an average GC content of 31%. The coding potential (for proteins and RNAs) was 80.9%-87.5%. Each consists of 63 genes and 5,422-9,600 bp non-coding sequences. The coding regions of the seven mt genomes were similar in size to those of *T. pseudonana* and *P. tricornutum*[28], yet the coding potential of *Nannochloropsis* mt genomes was higher, suggesting a relatively compact genome structure. Although most regions of the seven mt genomes were conserved, a pair of 3.5Kb-long, *cox1*-harboring repeats were found only in CCMP527 and CCMP537. Two segments of genes (*rps8*-*rpl6*-*rps2*-*rps4*, *rpl2*-*rps19*-*rps3*-*rpl16*) were conserved in previously reported stramenopiles including diatoms and brown algae. However in *Nannochloropsis*, the bacterial S10 operon block (*rpl2*-*rps19*-*rps3*-*rpl16*) was interrupted by *rpl22* which inserted between *rps19* and *rps3*.

Neither group I nor group II type introns were present in any of the *Nannochloropsis* organelle genes. Although the pt and mt genomes of CCMP529 and CCMP525 possessed increased numbers of small dispersed repetitive sequences compared to other *Nannochloropsis* pt and mt genomes, overall there were fewer repeats in the *Nannochloropsis* pt and mt genomes compared to those of diatoms. Moreover, the seven sets of pt and mt genomes were highly conserved in gene content and gene size (Figure 1A and B). In addition, the aligned regions (representing 96.89% and 97.16% of pt and mt genome lengths, respectively) showed high similarities (Figure 1C and D), with protein-coding regions generally more conserved than noncoding regions. Therefore, compactness in pt and mt genome organization is a shared feature among the seven *Nannochloropsis* strains.

### Protein complements of the organelle genomes

**Figure 2:**
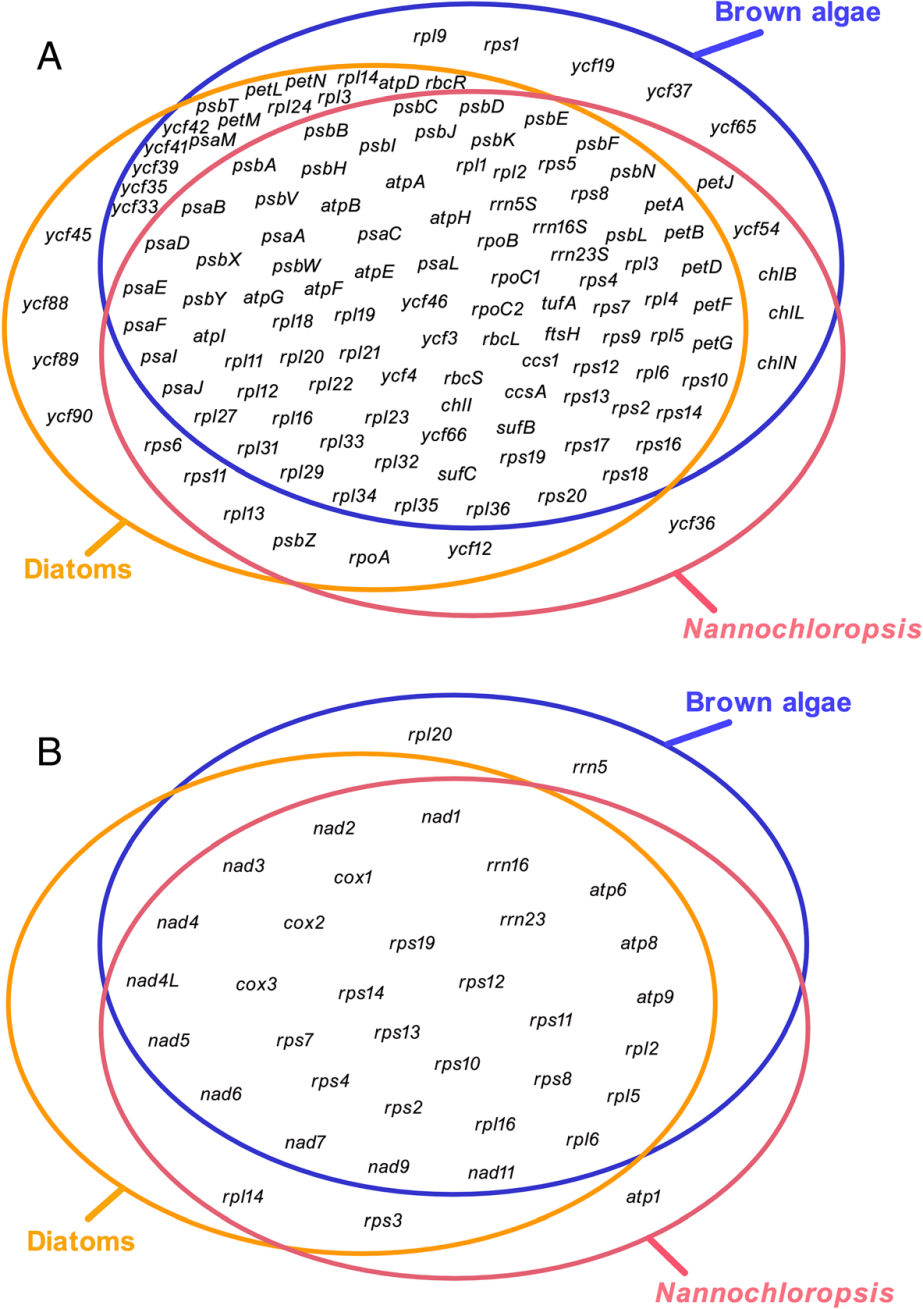
**Comparison of functional complements of organelle genomes.** Among *Nannochloropsis*, brown algae and diatoms, shared and lineage-specific genes from plastid and mitochondrial genomes are compared via Venn diagrams. **(A)** Shared and lineage-specific genes of different plastid genomes. **(B)** Shared and lineage-specific genes of different mitochondrial genomes.

Organelle genomes were thought to have undergone size- and functional reduction [29,30], and frequent genetic exchange via endosymbiotic gene transfer (EGT) and homologous recombination [31,32]. The present-day microalgal pt genomes mainly encode the components of photosystems, carbon assimilation, photosynthetic electron transport and gene translation machinery [33], while the mt genomes encode genes mostly involved in respiratory electron transport, oxidative phosphorylation, ATP synthesis and ribosome biosynthesis [5,34]. In *Nannochloropsis*, brown algae and diatoms, nearly all the photosystem I and photosystem II genes encoded by the pt genomes were retained in a high degree of consistency (Figure 2). However, a photosystem I gene (*psaM*) was lost in *Nannochloropsis* pt genome. A photosystem II gene (*psbM*) was also absent in the pt genomes of *Nannochloropsis* as in other red algae, but was present in the green algae lineage [35-39]. In addition, all of the cytochrome components found in other stramenopiles and the red lineage of algae (with the exception of *petL*) have been retained in *Nannochloropsis* pt genomes [40-46].

All of the ATP synthase genes (i.e.,*atpA*, *atpB*, *atpD*, *atpE*, *atpF*, *atpG*, *atpH* and *atpI*) were found in pt genomes of stramenopiles [8,26,47,48], except the seven *Nannochloropsis* strains in which *atpD* was missing. The chlorophyll biosynthesis genes *chlB*, *chlL* and *chlN* were believed to be transferred to nucleus via EGT in *Thalassiosira*, *Odontella* and *Heterosigma*[26,48], however four chlorophyll biosynthesis genes (*chlB*, *chlI*, *chlL* and *chlN*) are still present in the pt genomes of *Nannochloropsis*, *Ectocarpus*, *Fucus*, *Vaucheria* and *Aureoumbra* (Additional file 1: Table S1 and Figure 2) [8,47]. *RbcR* (*ycf30*), which was usually encoded by pt genomes and autonomously governs transcription of Rubisco operon in red algae [49], is present in either pt or nuclear genomes of all known stramenopiles except *Nannochloropsis.* The organization of pt ribosomal-protein genes in *Nannochloropsis* was also similar to that of *Thalassiosira*, *Odontella*, *Heterosigma*, *Ectocarpus* and *Fucus*, although *rpl9* and *rpl24* were lost in the *Nannochloropsis* pt genomes. In addition, *Synechococcus* phage S-SM2 gene segment was found in the *Nannochloropsis* pt genomes, which is likely a signature of their cyanobacterial origin.

To identify the functional distinction of *Nannochloropsis* mt genomes, the gene repertoires of 25 algal mt genomes were compared (Additional file 1: Table S2). The protein profiles of *Nannochloropsis* mt genomes are largely similar to those of *T. pseudonana* and *P. tricornutum*. However, *atp1* was retained only in *Nannochloropsis* mt genome (as are the cases in non-photosynthetic oomycetes such as *Phytophthora* spp. (another subgroup of stramenopiles) and *Saprolegnia ferax*) [50,51]. In *P. tricornutum* and *T. pseudonana*, *atp1* were thought to be transferred to the nuclear genome via endosymbiotic gene transfer [28]. Therefore *Nannochloropsis* exhibit an ancient feature, as is in the case of *Phytophthora*. On the other hand, the *rrn5* gene which encodes the 5S rRNA component was lost in *Nannochloropsis* and *Thalassiosira* mt genomes (but present in other stramenopiles such as *Heterosigma*, *Ectocarpus* and *Fucus*), suggesting structural diversity in mitochondrial translation systems of stramenopiles.

One prominent feature shaping organelle evolution is the targeting of certain nuclear-encoded proteins to organelles, which functionally complement the reduced gene content of pt/mt genomes [52]. Analysis of subcellular localization (with PredAlgo; [53]) of 9,756 putative proteins in IMET1 suggested that 973 and 1,620 proteins were targeted to mt and pt, respectively. They mainly include tRNA synthetases, ribosomal proteins, DNA polymerases, eukaryotic translation factors, transcription factors, TATA-box binding proteins and ATP synthases. These proteins might participate in the transcription and translation of organelle-encoding genes. In addition, 26 pentatricopeptide repeat-containing proteins (PPRs) were annotated, with six (g707, g1422, g2743, g3644, g3813 and g10257) targeting to mt and five (g2850, g3634, g3565, g8976 and g9207) to pt. In higher plants PPRs were likely involved in RNA editing, a process of post-transcriptional modification of RNA primary sequences through nucleotide deletion, insertion, or modification [54,55]. Thus in *Nannochloropsis* these proteins might participate in organelle RNA editing, which is an activity that has not been reported in microalgae.

### Evolution of organelle genomes

#### Organelle-based phylogeny of Nannochloropsis

Phylogenetic trees based on pt genomes were constructed by Maximum-Likelihood (ML), Maximum Parsimony (MP) and Neighbor-Joining (NJ) methods using a dataset of 39 conserved proteins (7,406 amino-acid positions) encoded by the pt genomes of four taxa of red algae and 13 green-algal taxa (the green clade*Viridiplantae* as outgroup; Additional file 1: Figure S1A). *Firstly*, red- and green-algal pt genomes respectively formed a distinct cluster. *Secondly*, within the red algae lineage, stramenopile species formed a monophyletic cluster. *Thirdly*, *Nannochloropsis* as a representative of Eustigmatophyte was closely related to the diatom *Thalassiosira*. Similar analysis of the mt genomes using a dataset of seven protein-coding genes (2,101 amino-acid positions) present in the lineages of green and red algae revealed that the stramenopile lineages were clustered despite weak support among *Nannochloropsis, Thalassiosira* and *Heterosigma* (Additional file 1: Figure S1B). Thus both pt and mt genomes suggested that *Nannochloropsis* are phylogenetically close to diatoms and brown algae.

#### Evolution of conserved coding regions in organelle genomes

In the coding regions of the seven pt genomes, 11,749 SNPs were identified (6,856 transitions, 4,871 transversions and 22 indels), representing a density of 152 SNPs/Kb (Additional file 1: Figure S2). Each of these 22 indels was a triplet of bases, which may not disrupt the open reading frames, reflecting a mechanism by which the cells fine-tune structure and function of encoded proteins. Among the SNPs, 8,845 were synonymous and 2,904 nonsynonymous, with a nonsynonymous/synonymous rate of 0.326.

In the coding region of the seven mt genomes, 4,990 SNPs (2,985 transitions, 1,997 transversions and 8 indels) were identified. The SNP density was 200 SNPs/Kb (Additional file 1: Figure S2), which is about 1.3 times higher than that of their pt counterparts. Similar to pt, indels in mt coding regions did not disrupt the open reading frames. Several parameters describing SNPs were similar between pt and mt, including SNP density (0.152 in pt and 0.200 in mt) and transition/transversion (1.408 in pt and 1.495 in mt).

To test the selection pressure of organelle protein-coding genes, ratio of nonsynonymous (Ka) versus synonymous substitution (Ks) was analyzed, which suggested a strong negative selection might have occurred in *Nannochloropsis* organelles. Ka/Ks of most pt genes were below 0.09 (except *psbK*, *psbN*, *psbW, atpF and ycf49*; Additional file 1: Figure S3A). Among the 38 mt-encoded genes, Ka/Ks were mostly no more than 0.1 (except *orf228*, *orf51*, *rps10*, *rpl5*, *atp8, rps14* and *orf321*, Additional file 1: Figure S3B). Notably, the mt o*rf228* (0.225) and pt *psbK* (0.098) were of the highest Ka/Ks ratios among all organelle genes. In *Nannochloropsis*, mean evolutionary rates of pt genes (at 0.031) and mt genes (at 0.064) were significantly lower than those of nuclear genes (at 0.093) (Additional file 1: Figure S3C; [56]), suggesting pt genomes have been evolving at a rate 50% and 33% of that of mt and nuclear genomes respectively.

### Hotspots of structural and sequence polymorphism in plastid and mitochondrial genomes

#### Hotspot of structural polymorphism in plastid genomes

**Figure 3:**
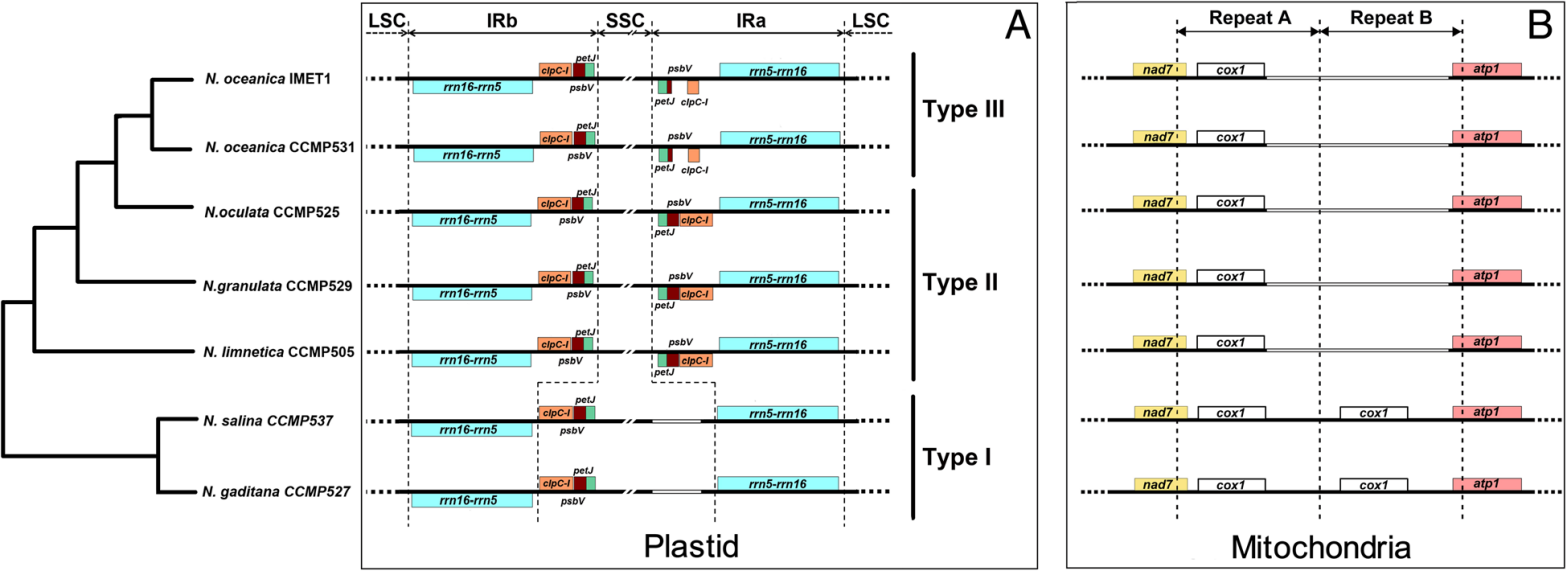
**Hotspots of structural polymorphism that drive the diversification of organelle genomes.** The phylogenetic tree on the left was based on whole-genome alignment of the seven complete mt genomes. **(A)** Structural and sequence polymorphism of inverted repeat in the pt genomes. **(B)** Structural and sequence polymorphism of the hotspot in the mt genomes. Within each sub-figure, genomic features were drawn proportionally to their actual length. Grey solid lines, inserted for alignment purposes, were not actual sequences.

Despite the slow evolution of the *Nannochloropsis* organelle genomes, a single hotspot of structural polymorphism was found in the pt genomes. A large inverted repeat (IR), as a canonical structure of pt genome, was present in the vast majority of higher plants and algae studied so far [57]. In many stramenopile algae such as *H. akashiwo, Thalassiosira oceanica and Skeletonema costatum*, the IRs are large in size (22 kb, 18 kb and 20 kb, respectively) and include 17 ~ 20 genes (including rRNA genes such as *rrn5*, *rrn16* and *rrn23*, ribosomal protein genes such as *rpl32*, *rpl21* and *rpl34*, and photosynthetic genes such as *psbA*, *psbY* and *psbC*; [48,58]). However, a pair of short IRs (IRa and IRb) each of 5,122 ~ 7,380 bp in size was found in each of the *Nannochloropsis* pt genomes (Figure 3), suggesting dramatic IR-size contraction. This may be due to the fewer number of genes harbored in the IRs: the ribosomal operon (*rrn5*, *rrn16* and *rrn23*) was present while ribosomal protein and photosystem genes were absent in each of the *Nannochloropsis* strains; moreover, *psbV*, *petJ* and *clpC-I* (which were absent in the IRs of diatoms and brown algae [8,26]) were present in only a subset of the strains (Figure 3A).

Interestingly, among the different *Nannochloropsis* species, evolutionary patterns of the two IRs (IRa and IRb; Figure 3A) were distinct: IRb were highly conserved, while IRa were extraordinarily hypervariable. There were three types of IRa in *Nannochloropsis* (Figure 3A): (i) Type I, found in CCMP527 and CCMP537, did not contain a region of *petJ*-*psbV*-*clpC-I*. (ii) Type II, found in CCMP529, CCMP525 and CCMP505, possessed a *petJ*-*psbV*-*clpC-I* that was an exact duplicate of that in IRb. (iii) Type III, present in CCMP531 and IMET1, encompassed a fragmented *petJ*-*psbV*-*clpC-I*, which differed from that in IRb due to a disruption of open reading frame (Additional file 1: Figure S4). The particular type of IRa that a given strain carries appeared to correlate with its specific lineage in *Nannochloropsis* genus, suggesting ancient IRa-diversifying events that likely have driven the speciation from the common ancestor of present-day *Nannochloropsis* strains.

Alignment of Type II and Type III IRa (in the five strains) revealed that the structural polymorphism leading to different IRa types was mainly due to hyper variation of sequences in two of the genes: *clpC-I_1* and *psbV_1.* Length of the two genes varied greatly as different start and stop codons were adopted among the strains (Additional file 1: Figure S4). Compared to Type II (CCMP529, CCMP525 and CCMP505), two bases were missing in Type III (IMET1 and CCMP531), resulting in a truncated *psbV_1*. Moreover, *clpC-I_1* ORFs were altered due to several intragenic insertions and deletions.

In the pt genome of higher plants, the border between SSC, LSC and IR exhibited a large degree of variation, in that many genes located in the junction are often lost and thus IR is reduced [59,60]. IR is important in higher plants because (1) it might stabilize ptDNA organizations [61], (2) it could mediate intra-molecular homologous recombination and (3) it may increase the relative copy number of rRNA genes [40]. The identification of a variable region located in the junction between IR and SSC in *Nannochloropsis* suggested an evolutionarily conserved link in hypervariable loci between higher plants and microalgae, however their differences are profound (Figure 3A): (i) Despite the presence of two IR copies in all higher plants and microalgae studied so far, the structural polymorphism is symmetric in higher plants (i.e., both IRs can be polymorphic; [57]) yet is strictly asymmetric in *Nannochloropsis*: only IRa were found as polymorphic while IRb were strictly conserved across all the seven strains tested. (ii) Unlike higher plants where the outer-most gene of IR underwent contraction [62,63], the internal gene of IR underwent contraction in *Nannochloropsis*. (iii) In higher plants the mechanism driving IR expansion/contraction was believed to be gene conversion and double-strand DNA breaks based on the observation of recombination points and tRNA duplication in IR [57,64]; however these observations were absent in any of the *Nannochloropsis* IR, suggesting a different and previously unappreciated mechanism for IR diversification in microalgae.

#### Single hotspot of structural polymorphism in mitochondrial genomes

A single hotspot of sequence variation was also discovered in mt genomes of the seven *Nannochloropsis* strains (Figure 1D). A pair of large repeats (~3,500 bp long), arranged as direct repeats, was found in *N. gaditana* CCMP527 and *N. salina* CCMP537. However only one such copy was present in each of the other strains (Figure 3B). Each of these regions was amplified by long-range PCR and fully sequenced to confirm the copy number variation.

Interestingly, in *N. gaditana* CCMP527 and *N. salina* CCMP537 mt genomes, each copy in the pair of large repeats harbors one intron-free *cox1* (encoding cytochrome *c* oxidase I). Such a duplication producing two 99%-identical copies of *cox1* was not found in either diatoms or brown algae. In diatom mt genomes (*Synedraacus*, *T. pseudonana* and *P. tricornutum*), a single copy of *cox1* (not found within repeats) contained ORFs-harboring introns [28,65], while in brown algae (*Dictyota dichotoma*, *Fucus vesiculosus* and *Desmarestia viridis*) a single intron-less *cox1* was present [66]. Therefore the observed direct repeats that harbor *cox1* was likely due to a duplication event in *Nannochloropsis* before the branching point of *N. gaditana* and *N. salina* (Figure 3B; the presence of two large repeats was also noted in *N. gaditana* CCMP526 mt genome [20]). Whether the event conveyed any biological consequence to the lineage is unknown.

### Strategy for sensitive and reliable intragenus phylotyping

#### Inter-species markers

Molecular markers (DNA barcoding) are a powerful taxonomy tool as compared to morphology-based classification [67]. The seven pairs of complete pt and mt genomes in *Nannochloropsis* enable the first exhaustive search and full assessment of organelle genes for introgenus phylotyping in microalgae. A pt (and mt) genome based reference phylogenetic tree was first constructed based on the concatenated nucleotide sequences of all protein-coding genes on the pt (and mt) genomes of the seven *Nannochloropsis* strains. Then each orthologous set of the intragenic and intergenic sequences from mt and pt genomes was extracted (a total of 230 individual regions) for construction of individual sequence based phylogenetic trees (Methods). Those orthologous sequence-sets consistent with the reference trees were analyzed further for their sensitivity and specificity in phylotyping. The Euclidean distance between two trees (each represented by one p-distance matrix) was used to quantify the similarity of the encoded phylogeny (Methods).

**Figure 4:**
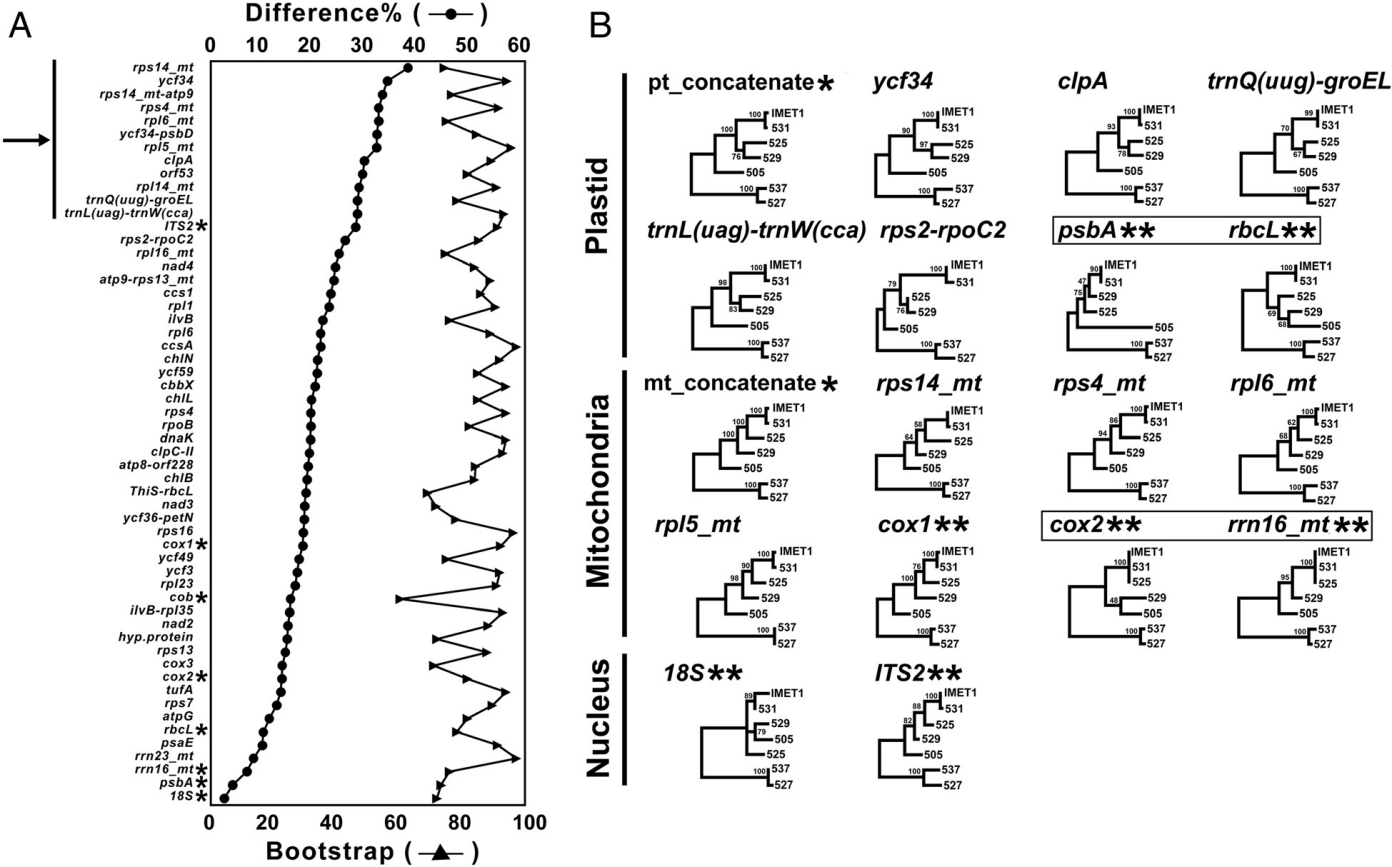
**New inter-species phylogenetic markers for *Nannochloropsis. *(A)** Comparison of the sensitivity and specificity among candidate regions/markers for inter-species phylogenetic reconstruction. The regions/markers derived from genic and intergenic sequences of pt and mt of the seven strains were listed on the X axe. The % nucleotide difference of each region/markers was calculated as the index for sensitivity. The average bootstrap values of all branches in the sequence-specific phylogenetic trees (maximum parsimony; MP) were shown as the index for specificity. *: presently used markers. Arrow: candidate phylogenetic markers that provided higher sensitivity than *ITS2.***(B)** Phylogeny of *Nannochloropsis* strains reconstructed from the presently used and our proposed sequence markers. Phylograms were derived using MP analysis. *: reference trees based on the concatenated sequences of all pt/mt protein-coding genes. **: presently used markers. Blank box: presently used markers whose phylogenetic reconstructions are not consistent with the reference trees. CCMP537 was assigned as the root for each of the trees.

A total of 54 candidate phylogenetic markers were identified whose nucleotide-sequence-based phylogenetic trees were consistent with the reference trees (Figure 4A). Forty-nine potential markers provided on average 1.5 times higher resolution (with SNP-density above 27%) than the seven commonly used phylogenetic markers (*ITS2*, *cox1, cob, cox2, rbcL, rrn16_mt and 18S*) in the interspecies taxonomy (Figure 4A; Table 2). Of these 49 candidates, twelve exhibited higher sensitivity than *ITS2*, which is the most commonly used microalgal phylogenetic marker at present and in effect provided the highest resolution among presently used phylogenetic markers in microalgae (Figure 4A; Table 2). Among these 12 markers, eight belonged to coding regions and another four to noncoding regions. Those encoded by mt included *rps14*, *rps4*, *rpl6*, *rpl5*, *orf53*, *rpl14* and *rps14-atp9* while those encoded by pt were *ycf34*, *clpA*, *ycf34-psbD*, *trnQ(uug)-groEL* and *trnL(uag)-trnW(cca)*. Among them, *rps14_mt* shows the highest resolution with interspecies difference of 37.71% and the Euclidean distance of 0.403 (Table 2), representing a sensitivity of 36.3% higher than *ITS2* (interspecies difference of 27.67%).

**Table 2:** **Comparison of candidate markers for interspecies phylotyping in *Nannochloropsis *genus**

**Gene**	**Origin**	**Size**	Difference*%		Euclidean distance***	SD****
			**Interspecies**	Intraspecies**		
*rps14_mt*	mt	297	37.71	0.34	0.397	0.046
*ycf34*	pt	252-261	33.72	0.00	0.409	0.038
*rps14_mt-atp9*	mt	102-195	32.83	0.00	0.500	0.048
*rps4_mt*	mt	726	32.09	0.41	0.260	0.031
*rpl6_mt*	mt	552	32.07	0.36	0.263	0.036
*ycf34-psbD*	pt	204-224	31.72	0.13	0.211	0.018
*rpl5_mt*	mt	525-540	31.67	0.57	0.299	0.037
*clpA*	pt	447-450	29.33	0.22	0.328	0.040
*orf53*	mt	156-162	29.01	0.00	0.258	0.041
*rpl14_mt*	mt	381	28.35	0.79	0.167	0.023
*trnQ(uug)-groEL*	pt	269-274	28.00	0.00	0.253	0.032
*trnL(uag)-trnW(cca)*	pt	648-673	27.99	0.46	0.234	0.021
*ITS2*	nc	385-499	27.67	0.52	-	-
*rps2-rpoC2*	pt	162-193	25.63	1.04	0.281	0.036
*rpl16_mt*	mt	432	24.54	0.00	0.098	0.018
*nad4*	mt	1578	23.76	0.63	0.038	0.008
*atp9-rps13_mt*	mt	215-233	23.50	0.43	0.107	0.022
*ccs1*	pt	1260-1272	22.90	0.00	0.111	0.010
*rpl1*	pt	687	22.56	0.00	0.114	0.012
*ilvB*	pt	1479	21.37	0.00	0.084	0.008
*rpl6*	pt	543	20.99	0.18	0.054	0.008
*ccsA*	pt	918-921	20.96	0.22	0.105	0.015
*chlN*	pt	1326-1335	20.37	0.00	0.062	0.008
*ycf59*	pt	1044	20.31	0.00	0.054	0.007
*cbbX*	pt	1011	19.88	0.10	0.053	0.008
*chlL*	pt	867	19.26	0.12	0.026	0.005
*rps4*	pt	627	19.14	0.00	0.029	0.005
*rpoB*	pt	3168	19.10	0.03	0.034	0.004
*dnaK*	pt	1809	19.02	0.11	0.030	0.004
*clpC-II*	pt	1155	18.87	0.09	0.020	0.004
*atp8-orf228*	mt	1294-1413	18.55	0.29	0.140	0.012
*chlB*	pt	1521-1524	18.37	0.00	0.023	0.005
*ThiS-rbcL*	pt	314-330	18.15	0.00	0.052	0.011
*nad3*	mt	369	17.89	0.27	0.108	0.009
*ycf36-petN*	pt	391-397	17.84	0.00	0.055	0.010
*rps16*	pt	255	17.65	0.00	0.119	0.025
*cox1*	mt	1521	17.55	0.13	0.144	0.018
*ycf49*	pt	294-297	16.84	0.00	0.035	0.007
*ycf3*	pt	504	16.47	0.00	0.059	0.009
*rpl23*	pt	360	16.11	0.00	0.062	0.007
*cob*	mt	1161	15.25	0.00	0.207	0.024
*ilvB-rpl35*	pt	504-512	15.04	0.00	0.091	0.019
*nad2*	mt	1482	14.71	0.34	0.039	0.005
*hyp.protein*	pt	645-699	14.57	0.14	0.086	0.009
*rps13*	pt	372	14.25	0.00	0.107	0.011
*cox3*	mt	813	13.65	0.12	0.221	0.021
*cox2*	mt	909	13.53	0.00	0.215	0.018
*tufA*	pt	1230-1275	13.36	0.08	0.067	0.009
*rps7*	pt	456-477	12.55	0.00	0.160	0.017
*atpG*	pt	477-483	11.18	0.00	0.171	0.014
*rbcL*	pt	1464	10.04	0.00	0.213	0.021
*psaE*	pt	204	9.80	0.00	0.199	0.017
*rrn23_mt*	mt	2235	8.18	0.18	0.365	0.038
*rrn16_mt*	mt	1491-1494	6.87	0.00	0.381	0.035
*psbA*	pt	1083	4.16	0.00	0.364	0.036
*18S*	nc	1790-1792	2.51	0.16	-	-

Note: “-” indicated that the gene was not encoded by the organelle genomes. *Difference = SNP/Size; **Difference between IMET1 and CCMP531; ***A measure of the similarity between two trees calculated from the two p-distance matrixes that each represents a phylogenetic tree; ****Square deviation of the corresponding p-distance between two matrixes.

Furthermore, these new sequence markers yielded a phylogeny consistent with the reference trees. Among those presently used markers, however, only *cox1* (but not *psbA*, *rbcL*, *cox2* and *rrn16_mt*) produced a phylogeny in consensus with the reference trees (Figure 4B). The *psbA*, *rbcL*, *cox2* and *rrn16_mt* are not suitable for distinguishing closely related species due to their low SNP-density (4.16%-13.53% among the six *Nannochloropsis* species; Table 2), especially among *N. oceanica* (IMET1 and CCMP531), *N.oculata* CCMP525 and *N.granulata* CCMP529 (SNP-density ranging from 0.64% to 3.74%). Thus the newly identified candidate markers may be more suitable than current markers for species classification in *Nannochloropsis*.

To test their wider applicability, these new candidate markers (*rps14*, *rps4*, *rpl6*, *rpl5*, *orf53*, *rpl14, ycf34* and *clpA*) were searched in available organelle genomes from other algal genera: they were rarely present in mt or pt genomes of the green lineage (e. g. *Chlamydomonas*, *Volvox* and *Dunaliella*). *ITS2* and 18S rRNA are universally found and widely used for species-level identification in higher plants and algae, however their resolution is limited as shown in this study. Moreover, being localized on the nuclear genomes, *ITS2* and 18S rRNA genes can become divergent paralogous copies as a result of incomplete concerted evolution and sexual incompatibility among individuals [68,69]. Our proposed new organelle markers provide certain advantages: higher discriminatory power, clonal modes of evolution and non-Mendelian inheritance [70,71]. Our analysis also suggested different microalgal lineages may require different sets of organelle marker genes for reliable and sensitive intragenus phylotyping.

#### Intra-species phylogenetic markers

Intraspecies divergence of microalgal genomes can be significant: despite their close phylogenetic relationship, the comparison of nuclear genomes revealed significant differences in coding sequences between the two *N. oceanica* strains IMET1 and CCMP531 (2.6% IMET1-specific genes; Methods). Therefore sensitive and reliable phylogenetic markers for intraspecies phylotyping are crucial. We tested the presently used markers and the candidate species-level markers identified above on the two *N. oceanica* strains IMET1 and CCMP531. All presently used phylogenetic markers were not sufficiently sensitive to distinguish IMET1 and CCMP531 due to their low SNP density (e.g. 0, 0, 2, 1 SNPs were respectively detected in *cox1, rbcL, 18S and ITS2*). In fact, between IMET1 and CCMP531, merely 87 and 129 SNPs were found in pt and mt genomes respectively. Moreover the SNP loci were physically distributed in a scattered manner, confounding their utilization via PCR followed by sequencing for phylogenetic analysis (Figure 1C, 1D).

**Figure 5:**
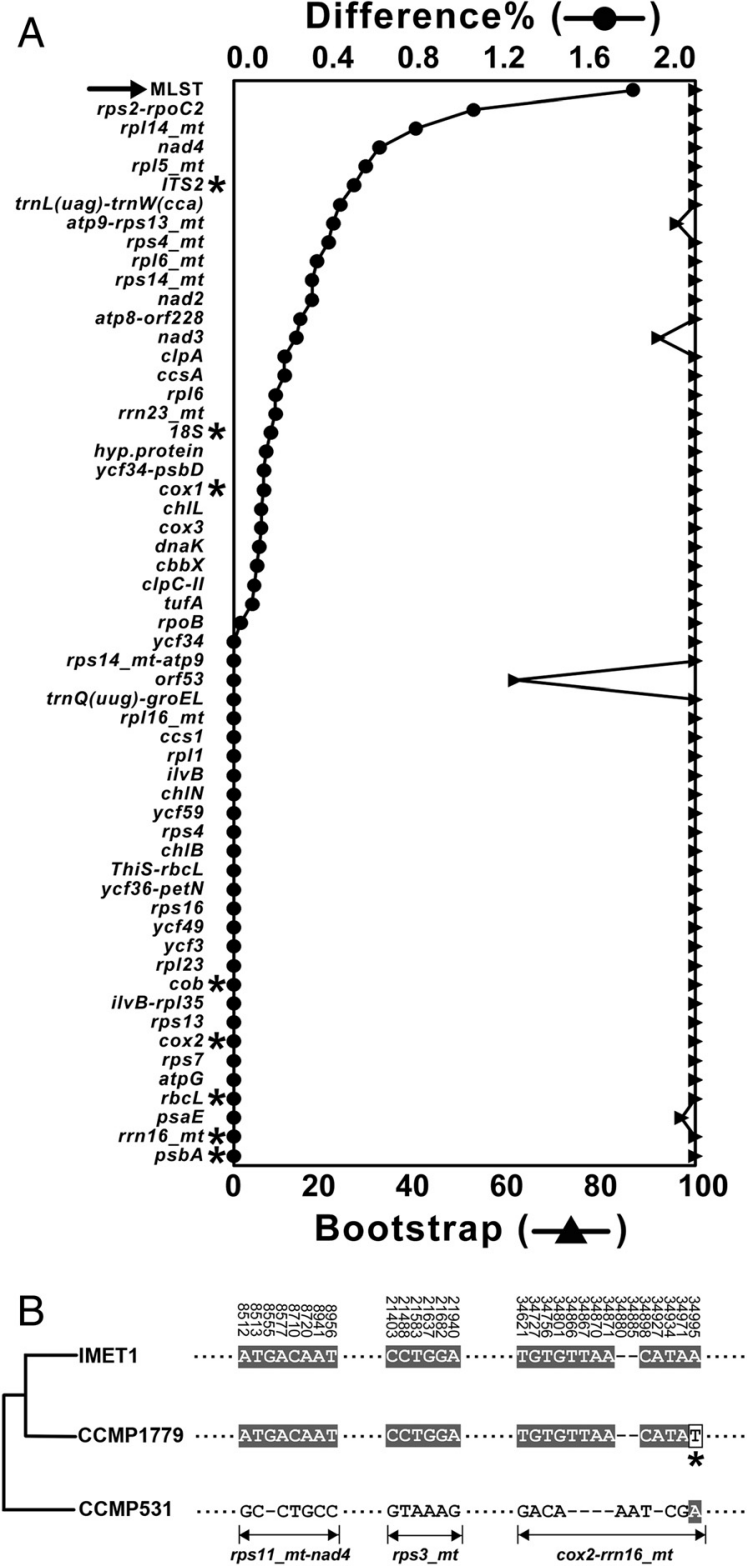
**Multiple locus sequence tag for high-resolution phylotyping of three closely related *N. oceanica *strains*. *(A)** Comparison of the sensitivity and specificity among candidate regions/markers for intra-species discrimination. The regions/markers derived from genic and intergenic sequences of pt and mt of the *N.oceanica* strains (IMET1 and CCMP531) were listed on the X axe. The % nucleotide difference of each region/markers was calculated as the index for sensitivity. The bootstrap values of the IMET1-CCMP531 branches in the sequence-specific phylogenetic trees (maximum parsimony; MP) were shown as the index for specificity. *: presently used markers. Arrow: the three regions (*rps11_mt*-*nad4*, *rps3_mt* and *cox2*-*rrn16_mt*) for Multiple-Locus Sequence Typing (MLST). **(B)** Phylogeny of IMET1, CCMP531 and CCMP1779 as reconstructed from three MLST loci. Grey background: identical loci in two strains. Blank box and *: A base that is different between CCMP1779 and IMET1.

To identify the most variable regions between IMET1 and CCMP531, the full lengths of IMET1 and CCMP531 organelle genomes were aligned. Only three highly variable regions (*rps11_mt*-*nad4*, *rps3_mt* and *cox2*-*rrn16_mt*) were found (Table 3), each with at least 5 SNPs per 1,000 bases. There were 8, 7 and 14 SNPs in *rps11*-*nad4*, *rps3* and *cox2*-*rrn16,* respectively and all these SNPs were synonymous substitutions. On IMET1 and CCMP531, the combined sequences of these three regions provided at least two-fold higher resolution than the above-mentioned presently used and new markers (Figure 5A). Thus combination of the three regions, as Multiple-Locus Sequence Typing (MLST) markers, can provide higher resolution for intraspecies discrimination.

**Table 3:** **Intraspecies phylogenetic markers of the three *Nannochloropsis oceanica *strains of CCMP531, CCMP1779 and IMET1**

**Region**	**Location**	**CCMP531**	**IMET1**	**CCMP1779**	**Synonymous/nonsynonymous**
*rps11_mt-nad4*	8512	G	A	A	-
	8513	C	T	T	-
	8555	-	G	G	-
	8577	C	A	A	-
*nad4*	8710	T	C	C	synonymous
	8720	G	A	A	synonymous
	8941	C	A	A	synonymous
	8956	C	T	T	synonymous
*rps3_mt*	21403	G	C	C	synonymous
	21488	T	C	C	synonymous
	21583	A	T	T	synonymous
	21637	A	G	G	synonymous
	21682	A	G	G	synonymous
	21940	G	A	A	synonymous
*cox2-rrn16_mt*	34621	G	T	T	-
	34727	A	G	G	-
	34756	C	T	T	-
	34801	A	G	G	-
	34866	-	T	T	-
	34867	-	T	T	-
	34870	-	A	A	-
	34871	-	A	A	-
	34880	A	-	-	-
	34885	A	-	-	-
	34898	T	C	C	-
	34927	-	A	A	-
	34934	C	T	T	-
	34971	G	A	A	-
	34995	A	A	T	-

Note: “-” indicated that the mutation was located at a non-protein-coding region.

To further test whether these MLST markers can be used for intraspecies phylogenetic reconstruction, we PCR-amplified and sequenced the three candidate MLST loci in CCMP1779, another *N.oceanica* strain whose nuclear genome along with partial plastid and mitochondrial genomes was recently released [21]. Despite significant divergence in the encoded proteome between IMET1 and CCMP1779 (1.8% IMET1-specific genes and 7.2% CCMP1779-specific genes; Methods), one of the presently used markers and our newly proposed *Nannochloropsis* species-level markers were able to discriminate the two strains. However, one high-quality SNP (confirmed by re-sequencing on both directions) was found in the *cox2-rrn16_mt* region of the IMET1 and CCMP1779 mt genomes. Thus our proposed MLST marker-set consisting of *rps11_mt*-*nad4*, *rps3_mt* and *cox2*-*rrn16_mt*, were able to discriminate the three closely related *N. oceanica* strains (Figure 5B). Moreover the reconstructed phylogeny based on the marker-set was consistent with that based on the whole-genome comparison (Figure 5B). These findings thus suggested a strategy for high-resolution intra-species typing of microalgae.

On the other hand, a total of 26 simple short repeats (SSRs; or microsatellites) were identified in the organelle genomes of IMET1 and CCMP531. Eleven of these SSRs were from pt genomes and 15 from mt genomes (Table 4). Between the two strains, 11 of the SSRs were shared. However two strain-specific SSRs were found in IMET1 pt genome: one poly (G) 14 mononucleotide intergenic sequence between *psbV* and *clpB* and one multiple (TA) 7 dinucleotide sequence located in *trnK(uuu)-trnG(gcc)*. Moreover a specific poly (A) 12 mononucleotide genic sequence located in *rps3* was found specifically in CCMP531 mt genome. SSRs offer potential advantage for strain discrimination as they are locus-specific, PCR-friendly and highly polymorphic [72]. Thus the three specific SSRs identified can be used to identify CCMP531 and IMET1. As SSR loci can be strain-specific, a searchable database of microalgal SSRs such as those reported here can be established for high-resolution microalgal strain-typing.

**Table 4:** Simple sequence repeat (SSRs) for intra-species discrimination

**Repeat**	**Length**	**Region**	**Locus**	**Organelle**	**Strain**
**A**	10	Intergenic	*psbB-petF*	pt	IMET1, 531
**T**	10	Genic	*rps12*	pt	IMET1, 531
**T**	10	Intergenic	*rpl16-rps3*	pt	IMET1, 531
**A**	10	Intergenic	*secA-rpl34*	pt	IMET1, 531
G^*^	14	Intergenic	*psbV-clpC*	pt	IMET1
TA^*^	14	Intergenic	*trnK(uuu)-trnG(gcc)*	pt	IMET1
T^*^	10	Intergenic	*tufA-rps7*	pt	531
**T**	11	Genic	*coxI*	mt	IMET1, 531
**A**	11	Genic	*atp1*	mt	IMET1, 531
**A**	10	Intergenic	*orf321*	mt	IMET1, 531
**A**	10	Genic	*rpl14*	mt	IMET1, 531
**T**	10	Intergenic	*trnD(gtc)-trnG(tcc)*	mt	IMET1, 531
**A**	10	Genic	*rps13*	mt	IMET1, 531
**T**	10	Intergenic	*trnK(ttt)-nad4L*	mt	IMET1, 531
A^*^	12	Genic	*rps3*	mt	531

^*^ SSRs that are specifically present in IMET1 or CCMP531.

## Conclusion

The complete organelle genome sequences of seven strains from six *Nannochloropsis* species enabled the first systematic analysis of organelle evolution within a microalgal genus. Both pt and mt genomes of *Nannochloropsis* were among the most compact known in stramenopiles, with the absence of introns, tight packaging of genes and a paucity of disperse repeats. Being highly conserved in gene content, gene size and gene order and strongly negatively selected in protein-coding regions, the pt and mt genomes were evolving at a rate 33% and 66%, respectively, of that occurred in nuclear genomes.

In *Nannochloropsis*, the pt genome diversification was driven by asymmetric evolution of two copies of inverted repeats (IRa and IRb), while mt genome evolution was shaped by a single evolution hotspot varied in copy-number of a 3.5Kb-long, *cox1*-harboring repeat. Genomic engineering of plastids, the primary energy production site in the cell, offers many opportunities to improve algal feedstock productivity. Transgene integration into a plastid genome may occur via homologous recombination of flanking sequences used in vectors. However, plastid transformation vectors are usually species-specifically designed, leading to low efficiency and even intractability in other species [73]. The high degree of conservation of pt and mt genomes suggested the feasibility of “universal vector” based on the highly conserved intergenic spacer regions. On the other hand, discovery of the evolutionary hotspots (i.e. IR in the pt genomes and the large repeats harboring *cox1* in the mt genomes) and the mechanism underlying the polymorphism should guide rational genetic engineering of plastids for possible phenotypic trait improvement and even for *de novo* design of organelle genomes for a synthetic algal cell [74].

This organelle phylogenome dataset, the most comprehensive for a microalgal genus to-date, also provided a first opportunity to evaluate existing phylogenetic reconstruction and strain-typing strategies in microalgae. Our analysis showed that, despite their wide uses in distinguishing among different microalgal genera, existing organelle gene markers (*cox1*, *cox2*, *psbA*, *rbcL* and *rrn16_mt*) and nuclear gene markers (*ITS2* and *18S*) have limited power in distinguishing closely related species due to the low SNP densities in these genes. Exhaustive searches and evaluation of all coding and non-coding sequences on the organelle genomes enabled us to propose the strategy for intra-genus phylotyping of microalgae: (i) twelve sequence markers of higher sensitivity than *ITS2* (the most widely used microalgal phylogenetic marker at present) for interspecies phylogeny, (ii) genus-specific multi-locus sequence tag of *rps11_mt*-*nad4*, *rps3_mt* and *cox2*-*rrn16_mt* for intraspecies phylogenetic analysis, and (iii) several SSR loci for reliable strain identification. As a result, new community resources such as databases of genus-specific phylogenetic markers and strain-identifier sequences (e.g. SSRs) should be developed for microalgae. The intragenus analysis strategy developed in this study may be generally applicable to other microalgal genera. As screening, development and protection of microalgae frequently demand species-, strain- and even isolate-level resolution, our findings may be valuable to the expanding algal biotechnology community.

## Methods

### Algal culture and genomic DNA extraction

*Nannochloropsis* strains including *N. oceanica* CCMP531, *N. salina* CCMP537, *N. gaditana* CCMP527, *N. oculata* CCMP525, *N. limnetica* CCMP505 and *N. granulata* CCMP529 were from the Provasoli-Guillard National Center for Culture of Marine Phytoplankton (CCMP). *Nannochloropsis oceanica* strain IMET1 was from the University of Maryland Biotechnology Institute. All of them were cultivated in liquid modified f/2 medium containing sterilized seawater (salinity1.5%, w/v) at 25°C under light–dark cycles of 12 h:12 h at an exposure intensity of 40 μmol/m^2^sec. Genomic DNA was then extracted via a published protocol [75].

### Sequencing and finishing of the 14 organelle genomes

All the organelle genomes were extracted from the whole-genome sequencing project of seven *Nannochloropsis* strains. *Firstly*, the high-quality draft genome sequence of *Nannochloropsis oceanica* strain IMET1 was generated using a hybrid sequencing and assembly strategy that combines the powers of pair-ended reads from 454 and Solexa. The pt and mt genomes of IMET1 were assembled from whole-genome shotgun reads using Newbler-v2.5.3 (Roche, Switzerland) and SOAPaligner-v2.21 [76] and then were manually finished using the Phred-Phrap-Consed package [77-79]. The IMET1 pt and mt sequences were circled into complete genomes with the support of high-quality reads. The IMET1 organelle genomes then served as a reference for assembly of other organelle genomes. *Secondly*, draft sequences of the other six *Nannochloropsis* strains were extracted from their whole-genome assemblies by blast using IMET1 sequence as a reference. Long Range PCR Kit (Takara) was employed using total genomic DNA as template to identify, confirm or bridge the gaps. Direction of single- and large-copy segments were also confirmed using PCR. Moreover the four junctions between the single-copy segments and inverted repeats were confirmed based on PCR product sequencing. Sequences from PCR products were assembled into the shotgun assemblies using CodonCode Aligner-v3.7.1 (CodonCodeCorp., USA).

### Sequence annotation and analysis

The organelle genomes were firstly annotated using DOGMA [80]. Genes not detected by DOGMA were identified by Blastx (http://www.ncbi.nlm.nih.gov/BLAST) and ORF Finder (http://www.ncbi.nlm.nih.gov/gorf). Ribosomal RNA genes were identified using RNAmmer [81]. Transfer RNA genes were identified using DOGMA and tRNAscan-SE 1.21 [82], and then confirmed by ERPIN [83] and TFAM Webserver-v1.3. Short repeat sequences including direct and inverted repeats in pt genome were discovered using REPuter [84] at repeat length of at least 30 bp and with a Hamming distance of 3. Tandem repeats were detected by Tandem Repeat Finder V4.0.4. Multiple sequence alignments of pt or mt genomes were performed via MEGA-v4.1-ClustalW [85]. Full alignments with annotations were visualized with VISTA [86]. The genetic divergence represented by p-distance was calculated by MEGA-v4.1. The circular gene maps of organelle genomes were drawn by GenomeVx [87] followed by manual modification.

### Phylogenetic analysis

To reconstruct whole-organelle based phylogeny, pt and mt datasets were assembled on the basis of genomes available in public databases and those newly sequenced in this study. Deduced amino acid sequences of each set of orthologous protein-coding genes were aligned using MUSCLE 3.7 (multiple sequence alignment by log-expectation) [88]. The ambiguously aligned regions in each alignment were removed and optimized using GBLOCKS 0.91b [89] with the -b2 option (minimal number of sequences for a flank position) set to 13. The concatenated protein alignments were used to infer phylogenetic trees using PhyML 2.4.4 [90] with the approximate likelihood ratio test [91]. Maximum Parsimony (MP) and Neighbor-Joining (NJ) analysis was performed with MEGA4.1 [85].

### Estimation of nucleotide substitution rate

A total of 37 mt and 110 pt protein-coding sequences among the seven *Nannochloropsis* strains were respectively aligned with MEGA-v4.1-ClustalW, using a maximum of 1,000 iterations for alignment refinement. Nonsynonymous substitutions rate (Ka), synonymous substitutions rate (Ks) and their ratio were estimated using the yn00 program of the PAML 4.4c [92] with the codon frequencies model F3 × 4 as substitution matrix. Ka and Ks were determined by the Nei-Gojobori method as implemented in yn00.

### Identification of phylogenetic markers

To mine the SNPs, the two sets (pt and mt) of genome sequences were respectively aligned with MEGA4.1-ClustalW. The SNPs were validated manually. To construct the phylogeny based on individual sequences, a total of 230 pt and mt coding and non-coding regions were employed to reconstruct phylogenetic trees by Maximum Parsimony (MP) via Phylip-v3.69 [93]. CCMP537 was assigned as the root for each of the trees. Then each of the sequence-based phylogenetic trees was individually compared with the corresponding pt or mt reference trees by Topd (TOPological Distance; [94]). The Euclidean distance of p-distance matrixes was used as the quantitative measure of the similarity between two trees (e.g. the test tree and the reference tree). Those trees consistent with reference trees were extracted to further analyze their power of discrimination.

To screen for intra-species markers for the *N. oceanica* strains, the organelle sequences of IMET1 and CCMP531 were aligned with MEGA4.1-ClustalW. Scatter diagram of variable-site distribution was drawn by DnaSP 4.10.7 [95], with a window length of 500 sites and a step size of 25 sites. Those sections with S-value of at least 6 were selected as highly variable regions. SNPs were validated by manual inspection and if necessary via targeted sequencing.

### Accession numbers

The complete sequences of the 14 plastid and mitochondrial genomes were deposited at GenBank: KC598086 and KC568456 for IMET1, KC598085 and KC568456 for CCMP529, KC598088 and KC568458 for CCMP537, KC598089 and KC568459 for CCMP505, KC598087 and KC568460 for CCMP525, KC598084 and KC568461 for CCMP527, and KC598090 and KC568462 for CCMP531.

## Competing interests

The authors declare that they have no competing interests.

## Authors’ contributions

JX, LW, YX, QH and FC designed and coordinated the study. DW and XJ contributed to whole genome sequencing project; LW, YX and JJ performed experiments; QZ, XS and KN participated in bioinformatics analysis; LW and YX carried out data analysis. LW, YX and JX wrote the manuscript. All authors read and approved the final manuscript.

## Supplementary Material

Additional file 1 Table S1 Comparison of gene contents in algal plastid genomes. **Table S2:** Comparison of gene contents in algal mitochondrial genomes. **Figure S1:** Whole-organelle-genome phylogeny of *Nannochloropsis.* All available mt and pt genomes of algae in public database to-date were included for the comparison. The trees were based on concatenated protein sequences encoded on pt **(A)** or mt **(B)**. Numbers on the internal nodes represent bootstrap values (≥50%) of Maximum-Likelihood (ML), Maximum Parsimony (MP) and Neighbor-Joining (NJ). **Figure S2:** Distribution of plastid **(A)** and mitochondrial **(B)** SNPs among the seven *Nannochloropsis* strains. **Figure S3:** The nonsynonymous (Ka) and synonymous (Ks) substitution rates of *Nannochloropsis* organelle genes. **(A)** Plastid genes. **(B)** Mitochondrial genes. **(C)** Comparison of sequence evolution rates among plastid, mitochondrial and nuclear genes. **Figure S4:** Fine-scale structural variation of plastid IRa among the *Nannochloropsis* strains. Insertions and deletions within the coding regions of *psbV* and *clpC* were shown. Dot: bases that are identical among the five strains. Grey background and dash: indels among the five strains. Blank box: protein-coding regions. Click here for file
