# Supplementary material for: Nannochloropsis plastid and mitochondrial phylogenomes reveal organelle diversification mechanism and intragenus phylotyping strategy in microalgae
Source: BMC Genomics. 2013 Aug 5;14:534. doi: 10.1186/1471-2164-14-534 (PMC3750441; doi:10.1186/1471-2164-14-534)

**Supplementary Tables and Figures**

**Table S1 Comparison of gene contents in microalgal plastid genomes**

|  | **Na** | **Tp** | **Os** | **Ha** | **Es** | **Fv** | **Ot** | **Vl** | **Al** | **Aa** | **Cm** | **Ppu** | **Eh** | **Rs** | **Sj** | **Vc** | **Ds** | **Cr** | **Pm** | **Pa** | **Pp** | **So** | **Ov** | **No** | **Cg** | **Mv** | **Cv** | **Ca** | **At** |
| --- | --- | --- | --- | --- | --- | --- | --- | --- | --- | --- | --- | --- | --- | --- | --- | --- | --- | --- | --- | --- | --- | --- | --- | --- | --- | --- | --- | --- | --- |
| **Photosystem I** |  |  |  |  |  |  |  |  |  |  |  |  |  |  |  |  |  |  |  |  |  |  |  |  |  |  |  |  |  |
| *psaA* | ■ | ■ | ■ | ■ | ■ | ■ | ■ | ■ | ■ | ■ | ■ | ■ | ■ | ■ |  | ■ | ■ | ■ | ■ | ■ | ■ | ■ | ■ | ■ | ■ | ■ | ■ | ■ | ■ |
| *psaB* | ■ | ■ | ■ | ■ | ■ | ■ | ■ | ■ | ■ | ■ | ■ | ■ | ■ | ■ |  | ■ | ■ | ■ | ■ | ■ | ■ | ■ | ■ | ■ | ■ | ■ | ■ | ■ | ■ |
| *psaC* | ■ | ■ | ■ | ■ | ■ | ■ | ■ | ■ | ■ | ■ | ■ | ■ | ■ | ■ |  | ■ | ■ | ■ | ■ | ■ | ■ | ■ | ■ | ■ | ■ | ■ | ■ | ■ | ■ |
| *psaD* | ■ | ■ | ■ | ■ | ■ | ■ |  | ■ | ■ | ■ | ■ | ■ | ■ | ■ |  |  |  |  |  |  |  |  |  |  |  |  |  |  |  |
| *psaE* | ■ | ■ | ■ | ■ | ■ | ■ |  | ■ |  |  | ■ | ■ |  | ■ |  |  |  |  |  |  |  |  |  |  |  |  |  |  |  |
| *psaF* | ■ | ■ | ■ | ■ | ■ | ■ |  | ■ | ■ |  | ■ | ■ | ■ | ■ |  |  |  |  |  |  |  |  |  |  |  |  |  |  |  |
| *psaI* | ■ | ■ | ■ | ■ | ■ | ■ | ■ |  |  |  | ■ | ■ | ■ | ■ |  |  |  |  | ■ | ■ | ■ |  | ■ | ■ | ■ | ■ | ■ | ■ | ■ |
| *psaJ* | ■ | ■ | ■ | ■ | ■ | ■ | ■ | ■ | ■ | ■ | ■ | ■ | ■ | ■ |  | ■ | ■ | ■ | ■ | ■ |  | ■ | ■ | ■ | ■ | ■ | ■ | ■ | ■ |
| *psaK* |  |  |  |  |  |  |  |  |  |  | ■ | ■ |  | ■ |  |  |  |  |  |  |  |  |  |  |  |  |  |  |  |
| *psaL* | ■ | ■ | ■ | ■ | ■ | ■ |  | ■ | ■ | ■ | ■ | ■ | ■ | ■ |  |  |  |  |  |  |  |  |  |  |  |  |  |  |  |
| *psaM* |  | ■ | ■ | ■ | ■ | ■ | ■ | ■ | ■ | ■ | ■ | ■ | ■ | ■ |  |  |  |  | ■ | ■ |  |  | ■ |  | ■ | ■ | ■ | ■ |  |

“■” indicate the presence of the gene on the particular plastid genome, while blank indicate absence. Na: *Nannochloropsis*, Tp: *Thalassiosira*, Os: *Odontella*, Ha: *Heterosigma*, Es: *Ectocarpus*, Fv: *Fucus*, Ot: *Ostreococcus*, Vl: *Vaucherialitorea*, Al: *Aureoumbra*, Aa: *Aureococcus*, Cm: *Cyanidioschyzon*, Ppu: *Porphyra*, Eh: *Emiliania*, Rs: *Rhodomonas*, Sj: *Simulium*, Vc: *Volvox*, Ds: *Dunaliella*, Cr: *Chlamydomonas*, Pm: *Pedinomonas*, Pa: *Pseudendoclonium*, Pp: *Pycnococcus*, So: *Scenedesmus*, Ov: *Oltmannsiellopsis*, No: *Nephroselmis*, Cg: *Chaetosphaeridium*, Mv: *Mesostigma*, Cv: *Chara*, Ca: *Chlorokybus*, At: *Arabidopsis*.

| **Photosystem II** |  |  |  |  |  |  |  |  |  |  |  |  |  |  |  |  |  |  |  |  |  |  |  |  |  |  |  |  |  |
| --- | --- | --- | --- | --- | --- | --- | --- | --- | --- | --- | --- | --- | --- | --- | --- | --- | --- | --- | --- | --- | --- | --- | --- | --- | --- | --- | --- | --- | --- |
| *psbA* | ■ | ■ | ■ | ■ | ■ | ■ | ■ | ■ | ■ | ■ | ■ | ■ | ■ | ■ |  | ■ | ■ | ■ | ■ | ■ | ■ | ■ | ■ | ■ | ■ | ■ | ■ | ■ | ■ |
| *psbB* | ■ | ■ | ■ | ■ | ■ | ■ | ■ | ■ | ■ | ■ | ■ | ■ | ■ | ■ |  | ■ | ■ | ■ | ■ | ■ | ■ | ■ | ■ | ■ | ■ | ■ | ■ | ■ | ■ |
| *psbC* | ■ | ■ | ■ | ■ | ■ | ■ | ■ | ■ | ■ | ■ | ■ | ■ | ■ | ■ |  | ■ | ■ | ■ | ■ | ■ | ■ | ■ | ■ | ■ | ■ | ■ | ■ | ■ | ■ |
| *psbD* | ■ | ■ | ■ | ■ | ■ | ■ | ■ | ■ | ■ | ■ | ■ | ■ | ■ | ■ |  | ■ | ■ | ■ | ■ | ■ | ■ | ■ | ■ | ■ | ■ | ■ | ■ | ■ | ■ |
| *psbE* | ■ | ■ | ■ | ■ | ■ | ■ | ■ | ■ | ■ | ■ | ■ | ■ | ■ | ■ |  | ■ | ■ | ■ | ■ | ■ | ■ | ■ | ■ | ■ | ■ | ■ | ■ | ■ | ■ |
| *psbF* | ■ | ■ | ■ | ■ | ■ | ■ | ■ | ■ | ■ | ■ | ■ | ■ | ■ | ■ |  | ■ | ■ | ■ | ■ | ■ | ■ | ■ | ■ | ■ | ■ | ■ | ■ | ■ | ■ |
| *psbH* | ■ | ■ | ■ | ■ | ■ | ■ | ■ | ■ | ■ | ■ | ■ | ■ | ■ | ■ |  | ■ | ■ | ■ | ■ | ■ | ■ | ■ | ■ | ■ | ■ | ■ | ■ | ■ | ■ |
| *psbI* | ■ | ■ | ■ | ■ | ■ | ■ | ■ | ■ | ■ | ■ | ■ | ■ | ■ | ■ |  | ■ | ■ | ■ | ■ | ■ | ■ | ■ | ■ | ■ | ■ | ■ | ■ | ■ | ■ |
| *psbJ* | ■ | ■ | ■ | ■ | ■ | ■ | ■ | ■ | ■ | ■ | ■ | ■ | ■ | ■ |  | ■ | ■ | ■ | ■ | ■ | ■ | ■ | ■ | ■ | ■ | ■ | ■ | ■ | ■ |
| *psbK* | ■ | ■ | ■ | ■ | ■ | ■ | ■ | ■ | ■ | ■ | ■ | ■ | ■ | ■ |  | ■ | ■ | ■ | ■ | ■ | ■ | ■ | ■ | ■ | ■ | ■ | ■ | ■ | ■ |
| *psbL* | ■ | ■ | ■ | ■ | ■ | ■ | ■ | ■ | ■ | ■ | ■ | ■ | ■ | ■ |  | ■ | ■ | ■ | ■ | ■ | ■ | ■ | ■ | ■ | ■ | ■ | ■ | ■ | ■ |
| *psbM* |  |  |  |  |  |  |  |  |  |  |  |  |  |  |  | ■ | ■ | ■ | ■ | ■ |  | ■ | ■ | ■ | ■ | ■ | ■ | ■ | ■ |
| *psbN* | ■ | ■ | ■ | ■ | ■ | ■ | ■ | ■ | ■ | ■ | ■ | ■ | ■ | ■ |  | ■ | ■ | ■ | ■ | ■ | ■ | ■ | ■ | ■ | ■ | ■ | ■ | ■ | ■ |
| *psbT* |  | ■ | ■ | ■ | ■ | ■ | ■ | ■ | ■ | ■ | ■ | ■ | ■ | ■ |  | ■ | ■ | ■ | ■ | ■ | ■ | ■ | ■ | ■ | ■ | ■ | ■ | ■ | ■ |
| *psbV* | ■ | ■ | ■ | ■ | ■ | ■ |  | ■ | ■ | ■ | ■ | ■ | ■ | ■ |  |  |  |  |  |  |  |  |  |  |  |  |  |  |  |
| *psbW(psb28)* | ■ | ■ | ■ | ■ | ■ | ■ |  | ■ |  |  | ■ | ■ |  | ■ |  |  |  |  |  |  |  |  |  |  |  |  |  |  |  |
| *psbX* | ■ | ■ | ■ | ■ | ■ | ■ |  | ■ | ■ | ■ | ■ |  |  | ■ |  |  |  |  |  |  |  |  |  |  |  |  | ■ |  |  |
| *psbY* | ■ | ■ | ■ | ■ | ■ | ■ |  | ■ |  |  | ■ |  |  | ■ |  |  |  |  |  |  |  |  |  |  |  |  |  |  |  |
| *psbZ(ycf9)* | ■ | ■ | ■ | ■ |  |  | ■ | ■ |  |  | ■ | ■ | ■ | ■ |  | ■ | ■ | ■ | ■ | ■ | ■ | ■ | ■ | ■ | ■ | ■ | ■ | ■ | ■ |
| **Cytochrome b6/F** |  |  |  |  |  |  |  |  |  |  |  |  |  |  |  |  |  |  |  |  |  |  |  |  |  |  |  |  |  |
| *petA* | ■ | ■ | ■ | ■ | ■ | ■ | ■ | ■ | ■ | ■ | ■ | ■ | ■ | ■ |  | ■ | ■ | ■ | ■ | ■ | ■ | ■ | ■ | ■ | ■ | ■ | ■ | ■ | ■ |
| *petB* | ■ | ■ | ■ | ■ | ■ | ■ | ■ | ■ | ■ | ■ | ■ | ■ | ■ | ■ |  | ■ | ■ | ■ | ■ | ■ | ■ | ■ | ■ | ■ | ■ | ■ | ■ | ■ | ■ |
| *petD* | ■ | ■ | ■ | ■ | ■ | ■ |  | ■ | ■ | ■ | ■ | ■ | ■ | ■ |  | ■ | ■ | ■ | ■ | ■ | ■ | ■ | ■ | ■ | ■ | ■ | ■ | ■ | ■ |
| *petF* | ■ | ■ | ■ | ■ | ■ | ■ |  | ■ |  |  | ■ | ■ |  | ■ |  |  |  |  |  |  |  |  |  |  |  |  |  |  |  |
| *petG* | ■ | ■ | ■ | ■ | ■ | ■ | ■ | ■ | ■ | ■ | ■ | ■ | ■ | ■ |  | ■ | ■ | ■ | ■ | ■ | ■ | ■ | ■ | ■ | ■ | ■ | ■ | ■ | ■ |
| *petJ* | ■ |  |  | ■ | ■ | ■ |  | ■ |  |  | ■ | ■ |  |  |  |  |  |  |  |  |  |  |  |  |  |  |  |  |  |
| *petL(ycf7)* |  | ■ | ■ | ■ | ■ | ■ |  | ■ | ■ | ■ | ■ | ■ | ■ | ■ |  | ■ | ■ | ■ | ■ | ■ | ■ | ■ | ■ | ■ | ■ | ■ | ■ | ■ | ■ |
| *petM(ycf31)* |  | ■ | ■ | ■ | ■ | ■ |  | ■ | ■ | ■ | ■ | ■ | ■ | ■ |  |  |  |  |  |  |  |  |  |  |  |  |  |  |  |
| *petN(ycf6)* |  | ■ | ■ | ■ | ■ | ■ |  | ■ | ■ | ■ | ■ | ■ | ■ | ■ |  |  |  |  |  |  | ■ |  |  | ■ | ■ | ■ | ■ | ■ | ■ |
| **ATP synthase** |  |  |  |  |  |  |  |  |  |  |  |  |  |  |  |  |  |  |  |  |  |  |  |  |  |  |  |  |  |
| *atpA* | ■ | ■ | ■ | ■ | ■ | ■ | ■ | ■ | ■ | ■ | ■ | ■ | ■ | ■ |  | ■ | ■ | ■ | ■ | ■ | ■ | ■ | ■ | ■ | ■ | ■ | ■ | ■ | ■ |
| *atpB* | ■ | ■ | ■ | ■ | ■ | ■ | ■ | ■ | ■ | ■ | ■ | ■ | ■ | ■ |  | ■ | ■ | ■ | ■ | ■ | ■ | ■ | ■ | ■ | ■ | ■ | ■ | ■ | ■ |
| *atpD* |  | ■ | ■ | ■ | ■ | ■ |  | ■ | ■ | ■ | ■ | ■ | ■ | ■ |  |  |  |  |  |  |  |  |  |  |  |  |  |  |  |
| *atpE(atpC)* | ■ | ■ | ■ | ■ | ■ | ■ | ■ | ■ | ■ | ■ | ■ | ■ | ■ | ■ |  | ■ | ■ | ■ | ■ | ■ | ■ | ■ | ■ | ■ | ■ | ■ | ■ | ■ | ■ |
| *atpF* | ■ | ■ | ■ | ■ | ■ | ■ | ■ | ■ | ■ | ■ | ■ | ■ | ■ | ■ |  | ■ | ■ | ■ | ■ | ■ | ■ | ■ | ■ | ■ | ■ | ■ | ■ | ■ | ■ |
| *atpG* | ■ | ■ | ■ | ■ | ■ | ■ |  | ■ | ■ | ■ | ■ | ■ | ■ | ■ |  |  |  |  |  |  |  |  |  |  |  |  |  |  |  |
| *atpH* | ■ | ■ | ■ | ■ | ■ | ■ | ■ | ■ | ■ | ■ | ■ | ■ | ■ | ■ |  | ■ | ■ | ■ | ■ | ■ | ■ | ■ | ■ | ■ | ■ | ■ | ■ | ■ | ■ |
| *atpI* | ■ | ■ | ■ | ■ | ■ | ■ | ■ | ■ | ■ | ■ | ■ | ■ | ■ | ■ |  | ■ | ■ | ■ | ■ | ■ | ■ | ■ | ■ | ■ | ■ | ■ | ■ | ■ | ■ |
| **Chlorophyll biosynthesis** | | | | | | | | | | | | | | | | | | | | | | | | | | | | | |
| *chlB* | ■ |  |  |  | ■ | ■ |  | ■ | ■ |  |  | ■ |  |  |  | ■ | ■ | ■ |  |  |  | ■ | ■ | ■ | ■ | ■ | ■ | ■ |  |
| *chlI* | ■ | ■ | ■ | ■ | ■ | ■ |  | ■ | ■ | ■ | ■ | ■ | ■ | ■ |  |  |  |  | ■ | ■ | ■ |  | ■ | ■ | ■ | ■ | ■ | ■ |  |
| *chlL* | ■ |  |  |  | ■ | ■ |  | ■ | ■ |  |  | ■ |  |  |  | ■ | ■ | ■ |  |  | ■ | ■ | ■ | ■ | ■ | ■ | ■ | ■ |  |
| *chlN* | ■ |  |  |  | ■ | ■ |  | ■ | ■ |  |  | ■ |  |  |  | ■ | ■ | ■ |  |  | ■ | ■ | ■ | ■ | ■ | ■ | ■ | ■ |  |
| **Rubisco** |  |  |  |  |  |  |  |  |  |  |  |  |  |  |  |  |  |  |  |  |  |  |  |  |  |  |  |  |  |
| *rbcL* | ■ | ■ | ■ | ■ | ■ | ■ | ■ | ■ | ■ | ■ | ■ | ■ | ■ | ■ |  | ■ | ■ | ■ | ■ | ■ | ■ | ■ | ■ | ■ | ■ | ■ | ■ | ■ | ■ |
| *rbcR(ycf30)* |  | ■ | ■ | ■ | ■ | ■ |  |  | ■ | ■ | ■ | ■ | ■ | ■ |  |  |  |  |  |  |  |  |  |  |  |  | ■ |  |  |
| *rbcS* | ■ | ■ | ■ | ■ | ■ | ■ |  | ■ | ■ | ■ | ■ | ■ | ■ | ■ |  |  |  |  |  |  |  |  |  |  |  |  |  |  |  |
| **RNA polymerase** |  |  |  |  |  |  |  |  |  |  |  |  |  |  |  |  |  |  |  |  |  |  |  |  |  |  |  |  |  |
| *rpoA* | ■ | ■ | ■ | ■ | ■ | ■ | ■ | ■ | ■ | ■ | ■ | ■ | ■ | ■ | ■ | ■ | ■ | ■ | ■ | ■ | ■ | ■ | ■ | ■ | ■ | ■ | ■ | ■ | ■ |
| *rpoB* | ■ | ■ | ■ | ■ | ■ | ■ | ■ | ■ | ■ | ■ | ■ | ■ | ■ | ■ | ■ | ■ | ■ | ■ | ■ | ■ |  | ■ | ■ | ■ | ■ | ■ | ■ | ■ | ■ |
| *rpoC1* | ■ | ■ | ■ | ■ | ■ | ■ | ■ | ■ | ■ | ■ | ■ | ■ | ■ | ■ | ■ | ■ | ■ | ■ | ■ | ■ | ■ | ■ | ■ | ■ | ■ | ■ | ■ | ■ | ■ |
| *rpoC2* | ■ | ■ | ■ | ■ | ■ | ■ | ■ | ■ | ■ | ■ |  | ■ | ■ | ■ | ■ | ■ | ■ | ■ | ■ | ■ | ■ | ■ | ■ | ■ | ■ | ■ | ■ | ■ | ■ |
| *rpoZ* |  |  |  |  |  |  |  |  |  |  | ■ |  |  |  |  |  |  |  |  |  |  |  |  |  |  |  |  |  |  |
| **NADH oxidoreductase** | | | | | | | | | | | | | | | | | | | | | | | | | | | | | |
| *ndhA* |  |  |  |  |  |  |  |  |  |  |  |  |  |  |  |  |  |  |  |  |  |  |  | ■ | ■ | ■ | ■ | ■ | ■ |
| *ndhB* |  |  |  |  |  |  |  |  |  |  |  |  |  |  |  |  |  |  |  |  |  |  |  | ■ | ■ | ■ | ■ | ■ | ■ |
| *ndhC* |  |  |  |  |  |  |  |  |  |  |  |  |  |  |  |  |  |  |  |  |  |  |  | ■ | ■ | ■ | ■ | ■ | ■ |
| *ndhD* |  |  |  |  |  |  |  |  |  |  |  |  |  |  |  |  |  |  |  |  |  |  |  | ■ | ■ | ■ | ■ | ■ | ■ |
| *ndhE* |  |  |  |  |  |  |  |  |  |  |  |  |  |  |  |  |  |  |  |  |  |  |  | ■ | ■ | ■ | ■ | ■ | ■ |
| *ndhF* |  |  |  |  |  |  |  |  |  |  |  |  |  |  |  |  |  |  |  |  |  |  |  | ■ | ■ | ■ | ■ | ■ | ■ |
| *ndhG* |  |  |  |  |  |  |  |  |  |  |  |  |  |  |  |  |  |  |  |  |  |  |  | ■ | ■ | ■ | ■ | ■ | ■ |
| *ndhH* |  |  |  |  |  |  |  |  |  |  |  |  |  |  |  |  |  |  |  |  |  |  |  | ■ | ■ | ■ | ■ | ■ | ■ |
| *ndhI* |  |  |  |  |  |  |  |  |  |  |  |  |  |  |  |  |  |  |  |  |  |  |  | ■ | ■ | ■ | ■ | ■ | ■ |
| *ndhJ* |  |  |  |  |  |  |  |  |  |  |  |  |  |  |  |  |  |  |  |  |  |  |  |  | ■ | ■ | ■ | ■ | ■ |
| *ndhK* |  |  |  |  |  |  |  |  |  |  |  |  |  |  |  |  |  |  |  |  |  |  |  | ■ | ■ | ■ | ■ | ■ | ■ |
| **LSU ribosomal proteins** | | | | | | | | | | | | | | | | | | | | | | | | | | | | | |
| *rpl1* | ■ | ■ | ■ | ■ | ■ | ■ |  | ■ | ■ | ■ | ■ | ■ |  | ■ |  |  |  |  |  |  |  |  |  |  |  |  |  |  |  |
| *rpl2* | ■ | ■ | ■ | ■ | ■ | ■ | ■ | ■ | ■ | ■ | ■ | ■ | ■ | ■ | ■ | ■ | ■ | ■ | ■ | ■ | ■ | ■ | ■ | ■ | ■ | ■ | ■ | ■ | ■ |
| *rpl3* | ■ | ■ | ■ | ■ | ■ | ■ |  | ■ | ■ | ■ | ■ | ■ | ■ | ■ |  |  |  |  |  |  |  |  |  |  |  |  |  |  |  |
| *rpl4* | ■ | ■ | ■ | ■ | ■ | ■ |  | ■ |  |  | ■ | ■ |  | ■ |  |  |  |  |  |  |  |  |  |  |  |  |  |  |  |
| *rpl5* | ■ | ■ | ■ | ■ | ■ | ■ | ■ | ■ | ■ | ■ | ■ | ■ | ■ | ■ | ■ | ■ | ■ | ■ | ■ | ■ | ■ | ■ | ■ | ■ | ■ | ■ | ■ | ■ |  |
| *rpl6* | ■ | ■ | ■ | ■ | ■ | ■ |  | ■ | ■ | ■ | ■ | ■ | ■ | ■ |  |  |  |  |  |  |  |  |  |  |  |  |  |  |  |
| *rpl9* |  |  |  |  | ■ | ■ |  | ■ |  |  |  | ■ |  |  |  |  |  |  |  |  |  |  |  |  |  |  |  |  |  |
| *rpl11* | ■ | ■ | ■ | ■ | ■ | ■ |  | ■ | ■ | ■ | ■ | ■ |  | ■ |  |  |  |  |  |  |  |  |  |  |  |  |  |  |  |
| *rpl12* | ■ | ■ | ■ | ■ | ■ | ■ |  | ■ |  |  | ■ | ■ |  | ■ | ■ |  |  |  | ■ | ■ |  | ■ | ■ | ■ | ■ |  |  |  |  |
| *rpl13* | ■ | ■ | ■ | ■ | ■ | ■ |  | ■ | ■ | ■ | ■ | ■ |  | ■ |  |  |  |  |  |  |  |  |  |  |  |  |  |  |  |
| *rpl14* | ■ | ■ | ■ | ■ | ■ | ■ | ■ | ■ | ■ | ■ | ■ | ■ | ■ | ■ | ■ | ■ | ■ | ■ | ■ | ■ | ■ | ■ | ■ | ■ | ■ | ■ | ■ | ■ | ■ |
| *rpl16* | ■ | ■ | ■ | ■ | ■ | ■ | ■ | ■ | ■ | ■ | ■ | ■ | ■ | ■ | ■ | ■ | ■ | ■ | ■ | ■ | ■ | ■ | ■ | ■ | ■ | ■ | ■ | ■ | ■ |
| *rpl18* | ■ | ■ | ■ | ■ | ■ | ■ |  | ■ |  |  | ■ | ■ |  | ■ |  |  |  |  |  |  |  |  |  |  |  |  |  |  |  |
| *rpl19* | ■ | ■ | ■ | ■ | ■ | ■ |  | ■ |  |  | ■ | ■ | ■ | ■ |  |  |  |  | ■ | ■ | ■ |  | ■ | ■ | ■ |  | ■ | ■ |  |
| *rpl20* | ■ | ■ | ■ | ■ | ■ | ■ | ■ | ■ | ■ | ■ | ■ | ■ | ■ | ■ | ■ | ■ | ■ | ■ | ■ | ■ | ■ | ■ | ■ | ■ | ■ | ■ | ■ | ■ | ■ |
| *rpl21* | ■ | ■ | ■ | ■ | ■ | ■ |  | ■ | ■ | ■ | ■ | ■ | ■ | ■ |  |  |  |  |  |  |  |  |  |  | ■ | ■ |  |  |  |
| *rpl22* | ■ | ■ | ■ | ■ | ■ | ■ |  | ■ | ■ | ■ | ■ | ■ | ■ | ■ |  |  |  |  |  |  |  |  |  |  | ■ | ■ | ■ | ■ | ■ |
| *rpl23* | ■ | ■ | ■ | ■ | ■ | ■ | ■ | ■ | ■ | ■ | ■ | ■ | ■ | ■ |  | ■ | ■ | ■ | ■ | ■ | ■ | ■ | ■ | ■ | ■ | ■ | ■ | ■ | ■ |
| *rpl24* |  | ■ | ■ | ■ | ■ | ■ |  | ■ | ■ | ■ | ■ | ■ |  | ■ |  |  |  |  |  |  |  |  |  |  |  |  |  |  |  |
| *rpl27* | ■ | ■ | ■ | ■ | ■ | ■ |  | ■ | ■ | ■ | ■ | ■ | ■ | ■ |  |  |  |  |  |  |  |  |  |  |  |  |  |  |  |
| *rpl28* |  |  |  |  |  |  |  |  |  |  | ■ | ■ |  |  |  |  |  |  |  |  |  |  |  |  |  |  |  |  |  |
| *rpl29* | ■ | ■ | ■ | ■ | ■ | ■ |  | ■ |  |  | ■ | ■ |  | ■ |  |  |  |  |  |  |  |  |  |  |  |  |  |  |  |
| *rpl31* | ■ | ■ | ■ | ■ | ■ | ■ |  | ■ | ■ | ■ | ■ | ■ | ■ | ■ |  |  |  |  |  |  |  |  |  |  |  |  |  |  |  |
| *rpl32* | ■ | ■ | ■ | ■ | ■ | ■ | ■ | ■ |  |  | ■ | ■ |  | ■ | ■ |  |  |  | ■ | ■ |  |  | ■ | ■ | ■ | ■ | ■ | ■ | ■ |
| *rpl33* | ■ | ■ | ■ | ■ | ■ | ■ |  | ■ | ■ | ■ | ■ | ■ | ■ | ■ |  |  |  |  |  |  |  |  |  |  | ■ | ■ | ■ | ■ | ■ |
| *rpl34* | ■ | ■ | ■ | ■ | ■ | ■ |  | ■ | ■ | ■ | ■ |  | ■ | ■ |  |  |  |  |  |  |  |  |  |  |  |  |  |  |  |
| *rpl35* | ■ | ■ | ■ | ■ | ■ | ■ |  | ■ | ■ | ■ | ■ | ■ |  | ■ |  |  |  |  |  |  |  |  |  |  |  |  |  |  |  |
| *rpl36* | ■ | ■ | ■ | ■ | ■ | ■ | ■ | ■ | ■ | ■ | ■ | ■ | ■ | ■ | ■ | ■ | ■ | ■ | ■ | ■ | ■ | ■ | ■ | ■ | ■ | ■ | ■ | ■ | ■ |
| **SSU ribosomal proteins** | | | | | | | | | | | | | | | | | | | | | | | | | | | | | |
| *rps1* |  |  |  | ■ | ■ | ■ |  | ■ |  |  | ■ | ■ |  |  |  |  |  |  |  |  |  |  |  |  |  |  |  |  |  |
| *rps2* | ■ | ■ | ■ | ■ | ■ | ■ | ■ | ■ | ■ | ■ | ■ | ■ | ■ | ■ |  | ■ | ■ | ■ | ■ | ■ | ■ | ■ | ■ | ■ | ■ | ■ | ■ | ■ | ■ |
| *rps3* | ■ | ■ | ■ | ■ | ■ | ■ | ■ | ■ | ■ | ■ | ■ | ■ | ■ | ■ | ■ | ■ | ■ | ■ | ■ | ■ | ■ | ■ | ■ | ■ | ■ | ■ | ■ | ■ | ■ |
| *rps4* | ■ | ■ | ■ | ■ | ■ | ■ | ■ | ■ | ■ | ■ | ■ | ■ | ■ | ■ | ■ | ■ | ■ | ■ | ■ | ■ | ■ | ■ | ■ | ■ | ■ | ■ | ■ | ■ | ■ |
| *rps5* | ■ | ■ | ■ | ■ | ■ | ■ |  | ■ | ■ | ■ | ■ | ■ | ■ | ■ |  |  |  |  |  |  |  |  |  |  |  |  |  |  |  |
| *rps6* | ■ | ■ | ■ |  |  |  |  |  | ■ | ■ | ■ | ■ | ■ | ■ |  |  |  |  |  |  |  |  |  |  |  |  |  |  |  |
| *rps7* | ■ | ■ | ■ | ■ | ■ | ■ | ■ | ■ | ■ | ■ | ■ | ■ | ■ | ■ | ■ | ■ | ■ | ■ | ■ | ■ | ■ | ■ | ■ | ■ | ■ | ■ | ■ | ■ | ■ |
| *rps8* | ■ | ■ | ■ | ■ | ■ | ■ | ■ | ■ | ■ | ■ | ■ | ■ | ■ | ■ | ■ | ■ | ■ | ■ | ■ | ■ | ■ | ■ | ■ | ■ | ■ | ■ | ■ | ■ | ■ |
| *rps9* | ■ | ■ | ■ | ■ | ■ | ■ | ■ | ■ | ■ | ■ | ■ | ■ | ■ | ■ |  | ■ | ■ | ■ | ■ | ■ |  | ■ | ■ | ■ |  |  | ■ | ■ |  |
| *rps10* | ■ | ■ | ■ | ■ | ■ | ■ |  | ■ | ■ | ■ | ■ | ■ | ■ | ■ |  |  |  |  |  |  |  |  |  |  |  |  |  |  |  |
| *rps11* | ■ | ■ | ■ | ■ | ■ | ■ | ■ | ■ | ■ | ■ | ■ | ■ | ■ | ■ | ■ | ■ | ■ | ■ | ■ | ■ | ■ | ■ | ■ | ■ | ■ | ■ | ■ | ■ | ■ |
| *rps12* | ■ | ■ | ■ | ■ | ■ | ■ | ■ | ■ | ■ | ■ | ■ | ■ | ■ | ■ | ■ | ■ | ■ | ■ | ■ | ■ | ■ | ■ | ■ | ■ | ■ | ■ | ■ | ■ | ■ |
| *rps13* | ■ | ■ | ■ | ■ | ■ | ■ |  | ■ | ■ | ■ | ■ | ■ | ■ | ■ |  |  |  |  |  |  |  |  |  |  |  |  |  |  |  |
| *rps14* | ■ | ■ | ■ | ■ | ■ | ■ | ■ | ■ | ■ | ■ | ■ | ■ | ■ | ■ | ■ | ■ | ■ | ■ | ■ | ■ | ■ | ■ | ■ | ■ | ■ | ■ | ■ | ■ | ■ |
| *rps15* |  |  |  |  |  |  |  |  |  |  |  |  |  |  |  |  |  |  |  |  |  |  |  |  |  | ■ | ■ | ■ | ■ |
| *rps16* | ■ | ■ | ■ | ■ | ■ | ■ |  | ■ | ■ | ■ | ■ | ■ | ■ | ■ |  |  |  |  |  |  |  |  |  |  | ■ | ■ | ■ | ■ | ■ |
| *rps17* | ■ | ■ | ■ | ■ | ■ | ■ |  | ■ | ■ | ■ | ■ | ■ | ■ | ■ |  |  |  |  |  |  |  |  |  |  |  |  |  |  |  |
| *rps18* | ■ | ■ | ■ | ■ | ■ | ■ | ■ | ■ | ■ | ■ | ■ | ■ | ■ | ■ |  | ■ | ■ | ■ | ■ | ■ | ■ | ■ | ■ | ■ | ■ | ■ | ■ | ■ | ■ |
| *rps19* | ■ | ■ | ■ | ■ | ■ | ■ | ■ | ■ | ■ | ■ | ■ | ■ | ■ | ■ | ■ | ■ | ■ | ■ | ■ | ■ | ■ | ■ | ■ | ■ | ■ | ■ | ■ | ■ | ■ |
| *rps20* | ■ | ■ | ■ | ■ | ■ | ■ |  | ■ |  |  | ■ | ■ |  | ■ |  |  |  |  |  |  |  |  |  |  |  |  |  |  |  |
| **Translation factors** |  |  |  |  |  |  |  |  |  |  |  |  |  |  |  |  |  |  |  |  |  |  |  |  |  |  |  |  |  |
| *infA* |  |  |  |  |  |  | ■ |  |  |  |  |  |  |  |  |  |  |  |  | ■ | ■ | ■ | ■ | ■ | ■ | ■ | ■ | ■ |  |
| *infB* |  |  |  |  |  |  |  |  |  |  | ■ | ■ |  | ■ |  |  |  |  |  |  |  |  |  |  |  |  |  |  |  |
| *tufA* | ■ | ■ | ■ | ■ | ■ | ■ | ■ | ■ | ■ | ■ | ■ | ■ | ■ | ■ | ■ | ■ | ■ | ■ | ■ | ■ | ■ | ■ | ■ | ■ | ■ | ■ | ■ | ■ |  |
| **Division** |  |  |  |  |  |  |  |  |  |  |  |  |  |  |  |  |  |  |  |  |  |  |  |  |  |  |  |  |  |
| *ftsH(ycf25)* | ■ | ■ | ■ | ■ | ■ | ■ |  | ■ |  |  | ■ | ■ |  | ■ | ■ | ■ | ■ |  | ■ | ■ | ■ | ■ | ■ |  | ■ | ■ | ■ | ■ |  |
| *fstI* |  |  |  |  |  |  |  |  |  |  |  |  |  |  |  |  |  |  |  |  |  |  |  | ■ |  |  | ■ | ■ |  |
| *ftsW* |  |  |  |  |  |  |  |  |  |  |  |  |  |  |  |  |  |  |  |  |  |  |  | ■ |  |  | ■ | ■ |  |
| *minD* |  |  |  |  |  |  |  |  |  |  |  |  | ■ | ■ |  |  |  |  | ■ | ■ |  |  | ■ | ■ |  |  | ■ | ■ |  |
| *minE* |  |  |  |  |  |  |  |  |  |  |  |  |  | ■ |  |  |  |  |  |  |  |  |  |  |  |  |  |  |  |
| **Miscellaneous proteins** | | | | | | | | | | | | | | | | | | | | | | | | | | | | | |
| *accA* |  |  |  |  |  |  |  |  |  |  | ■ | ■ |  |  |  |  |  |  |  |  |  |  |  |  |  |  |  |  |  |
| *accB* |  |  |  |  |  |  |  |  |  |  | ■ | ■ |  |  |  |  |  |  |  |  |  |  |  |  |  |  |  |  |  |
| *accD* |  |  |  |  |  |  |  |  |  |  | ■ | ■ |  |  | ■ |  |  |  | ■ | ■ |  |  | ■ | ■ | ■ | ■ | ■ |  | ■ |
| *cemA(ycf10)* |  |  |  |  |  |  |  |  |  |  |  | ■ |  | ■ |  | ■ | ■ | ■ | ■ | ■ | ■ | ■ | ■ | ■ | ■ | ■ | ■ | ■ | ■ |
| *clpC* | ■ | ■ | ■ | ■ | ■ | ■ |  | ■ | ■ | ■ | ■ | ■ | ■ | ■ |  |  |  |  |  |  |  |  |  |  |  |  |  |  |  |
| *clpP* |  |  |  |  |  |  |  |  |  |  |  |  |  |  |  | ■ | ■ | ■ | ■ | ■ | ■ | ■ | ■ | ■ | ■ | ■ | ■ | ■ | ■ |
| *ccs1(ycf44)* | ■ | ■ | ■ | ■ | ■ | ■ |  | ■ | ■ | ■ | ■ | ■ | ■ | ■ |  |  |  |  |  |  |  |  |  |  |  |  |  |  |  |
| *ccsA(ycf5)* | ■ | ■ | ■ | ■ | ■ | ■ |  | ■ | ■ | ■ | ■ | ■ | ■ | ■ |  | ■ | ■ | ■ | ■ | ■ |  | ■ | ■ | ■ | ■ | ■ | ■ | ■ | ■ |
| *cysA* |  |  |  |  |  |  |  |  |  |  |  |  |  |  |  |  |  |  | ■ |  |  |  |  | ■ |  |  | ■ | ■ |  |
| *cyst* |  |  |  |  |  |  |  |  |  |  | ■ |  |  |  | ■ |  |  |  | ■ |  | ■ |  |  | ■ |  |  | ■ | ■ |  |
| **Conserved proteins** |  |  |  |  |  |  |  |  |  |  |  |  |  |  |  |  |  |  |  |  |  |  |  |  |  |  |  |  |  |
| *ycf1* |  |  |  |  |  |  |  |  |  |  |  |  |  |  | ■ | ■ | ■ |  | ■ | ■ | ■ | ■ | ■ | ■ | ■ | ■ | ■ | ■ | ■ |
| *ycf2* |  |  |  |  |  |  |  |  |  |  |  |  |  |  |  |  |  |  |  |  |  |  |  | ■ |  |  |  |  | ■ |
| *ycf3* | ■ | ■ | ■ | ■ | ■ | ■ | ■ | ■ | ■ | ■ | ■ | ■ | ■ | ■ |  | ■ | ■ | ■ | ■ | ■ | ■ | ■ | ■ | ■ | ■ | ■ | ■ | ■ | ■ |
| *ycf4* | ■ | ■ | ■ | ■ | ■ | ■ |  | ■ | ■ | ■ | ■ | ■ | ■ | ■ |  | ■ | ■ | ■ | ■ | ■ | ■ | ■ | ■ | ■ | ■ | ■ | ■ | ■ | ■ |
| *ycf12* | ■ | ■ | ■ | ■ | ■ | ■ | ■ | ■ | ■ | ■ | ■ | ■ | ■ | ■ |  | ■ | ■ | ■ | ■ | ■ | ■ |  | ■ | ■ | ■ | ■ | ■ | ■ |  |
| *ycf16(sufC)* | ■ | ■ | ■ | ■ | ■ | ■ |  | ■ |  |  | ■ | ■ |  | ■ |  |  |  |  |  |  |  |  |  |  |  |  |  |  |  |
| *ycf18* |  |  |  |  |  |  |  |  |  |  |  | ■ |  |  |  |  |  |  |  |  |  |  |  |  |  |  |  |  |  |
| *ycf19* |  |  |  |  | ■ | ■ |  | ■ |  |  | ■ | ■ | ■ | ■ |  |  |  |  |  |  |  |  |  |  |  |  |  |  |  |
| *ycf20* |  |  |  |  |  |  |  |  |  |  | ■ | ■ | ■ |  |  |  |  |  | ■ | ■ |  |  | ■ |  | ■ |  | ■ | ■ |  |
| *ycf21* |  |  |  |  |  |  |  |  |  |  |  | ■ |  |  |  |  |  |  |  |  |  |  |  |  |  |  |  |  |  |
| *ycf22* |  |  |  |  |  |  |  |  |  |  | ■ | ■ |  |  |  |  |  |  |  |  |  |  |  |  |  |  |  |  |  |
| *ycf23* |  |  |  |  |  |  |  |  |  |  | ■ | ■ |  |  |  |  |  |  |  |  |  |  |  |  |  |  |  |  |  |
| *ycf24(sufB)* | ■ | ■ | ■ | ■ | ■ | ■ |  | ■ | ■ | ■ | ■ | ■ | ■ | ■ |  |  |  |  |  |  |  |  |  |  |  |  |  |  |  |
| *ycf26* |  |  |  |  |  |  |  |  |  |  |  | ■ |  | ■ |  |  |  |  |  |  |  |  |  |  |  |  |  |  |  |
| *ycf28* |  |  |  |  |  |  |  |  |  |  | ■ | ■ |  |  |  |  |  |  |  |  |  |  |  |  |  |  |  |  |  |
| *ycf29* |  |  |  |  |  |  |  |  |  |  | ■ | ■ |  | ■ |  |  |  |  |  |  |  |  |  |  |  |  |  |  |  |
| *ycf32* |  |  |  |  |  |  |  |  |  |  |  | ■ |  |  |  |  |  |  |  |  |  |  |  |  |  |  |  |  |  |
| *ycf33(Escp43)* |  | ■ | ■ | ■ | ■ | ■ |  | ■ | ■ | ■ | ■ | ■ |  | ■ |  |  |  |  |  |  |  |  |  |  |  |  |  |  |  |
| *ycf34* |  |  |  | ■ | ■ | ■ |  |  |  |  |  | ■ |  |  |  |  |  |  |  |  |  |  |  |  |  |  |  |  |  |
| *ycf35* |  | ■ | ■ | ■ | ■ | ■ |  |  | ■ | ■ |  | ■ | ■ | ■ |  |  |  |  |  |  |  |  |  |  |  |  |  |  |  |
| *ycf36* | ■ |  |  | ■ |  |  |  | ■ |  |  |  | ■ |  | ■ |  |  |  |  |  |  |  |  |  |  |  |  |  |  |  |
| *ycf37* |  |  |  |  | ■ | ■ |  | ■ | ■ | ■ |  | ■ |  | ■ |  |  |  |  |  |  |  |  |  |  |  |  |  |  |  |
| *ycf38* |  |  |  |  |  |  |  |  |  |  | ■ | ■ |  |  |  |  |  |  |  |  |  |  |  |  |  |  |  |  |  |
| *ycf39* |  | ■ | ■ | ■ | ■ | ■ |  | ■ | ■ | ■ | ■ | ■ | ■ | ■ |  |  |  |  |  |  |  |  |  |  |  |  |  |  |  |
| *ycf41(Escp41)* |  | ■ | ■ | ■ | ■ | ■ |  | ■ |  |  |  |  |  |  |  |  |  |  |  |  |  |  |  |  |  |  |  |  |  |
| *ycf42* |  | ■ | ■ | ■ | ■ | ■ |  | ■ |  |  |  |  |  |  |  |  |  |  |  |  |  |  |  |  |  |  |  |  |  |
| *ycf45* |  | ■ | ■ | ■ |  |  |  |  | ■ |  |  |  | ■ |  |  |  |  |  |  |  |  |  |  |  |  |  |  |  |  |
| *ycf46(ORF491)* | ■ | ■ | ■ | ■ | ■ | ■ |  |  | ■ |  |  | ■ | ■ | ■ |  |  |  |  |  |  |  |  |  |  |  |  |  |  |  |
| *ycf49* |  |  |  |  |  |  |  |  |  |  | ■ |  |  |  |  |  |  |  |  |  |  |  |  |  |  |  |  |  |  |
| *ycf52* |  |  |  |  |  |  |  |  |  |  | ■ |  |  |  |  |  |  |  |  |  |  |  |  |  |  |  |  |  |  |
| *ycf53* |  |  |  |  |  |  |  |  |  |  | ■ |  |  |  |  |  |  |  |  |  |  |  |  |  |  |  |  |  |  |
| *ycf54* | ■ |  |  | ■ | ■ | ■ |  | ■ |  |  | ■ |  |  |  |  |  |  |  |  |  |  |  |  |  |  |  |  |  |  |
| *ycf55* |  |  |  |  |  |  |  |  |  |  | ■ |  | ■ |  |  |  |  |  |  |  |  |  |  |  |  |  |  |  |  |
| *ycf60* |  |  |  |  |  |  |  | ■ |  |  | ■ |  | ■ |  |  |  |  |  |  |  |  |  |  |  |  |  |  |  |  |
| *ycf61* |  |  |  |  |  |  |  |  |  |  |  | ■ |  | ■ |  |  |  |  |  |  |  |  |  |  |  |  | ■ | ■ |  |
| *ycf62* |  |  |  |  |  |  |  |  |  |  | ■ |  |  |  |  |  |  |  |  | ■ |  |  |  | ■ | ■ | ■ | ■ | ■ |  |
| *ycf65(psrp3)* |  |  |  | ■ | ■ | ■ |  | ■ |  |  | ■ | ■ | ■ | ■ |  |  |  |  |  |  |  |  |  |  |  |  | ■ | ■ |  |
| *ycf66* | ■ | ■ |  | ■ | ■ | ■ |  | ■ |  |  |  |  |  |  |  |  |  |  |  |  |  |  |  |  | ■ | ■ | ■ | ■ |  |
| *ycf80* |  |  |  |  |  |  |  |  |  |  | ■ |  | ■ |  |  |  |  |  |  |  |  |  |  |  |  |  |  |  |  |
| *ycf81* |  |  |  |  |  |  |  |  |  |  |  |  |  |  |  |  |  |  |  |  |  |  |  | ■ |  |  |  | ■ |  |
| *ycf82* |  |  |  |  |  |  |  |  |  |  | ■ |  |  |  |  |  |  |  |  |  |  |  |  |  |  |  |  |  |  |
| *ycf83* |  |  |  |  |  |  |  |  |  |  | ■ |  |  |  |  |  |  |  |  |  |  |  |  |  |  |  |  |  |  |
| *ycf84* |  |  |  |  |  |  |  |  |  |  | ■ |  |  |  |  |  |  |  |  |  |  |  |  |  |  |  |  |  |  |
| *ycf85* |  |  |  |  |  |  |  |  |  |  | ■ |  |  |  |  |  |  |  |  |  |  |  |  |  |  |  |  |  |  |
| *ycf86* |  |  |  |  |  |  |  |  |  |  | ■ |  |  |  |  |  |  |  |  |  |  |  |  |  |  |  |  |  |  |
| *ycf88* |  | ■ |  |  |  |  |  |  |  |  |  |  |  |  |  |  |  |  |  |  |  |  |  |  |  |  |  |  |  |
| *ycf89* |  | ■ |  |  |  |  |  |  |  |  |  |  |  |  |  |  |  |  |  |  |  |  |  |  |  |  |  |  |  |
| *ycf90* |  | ■ |  |  |  |  |  |  |  |  |  |  |  |  |  |  |  |  |  |  |  |  |  |  |  |  |  |  |  |
| **Ribosomal RNAs** |  |  |  |  |  |  |  |  |  |  |  |  |  |  |  |  |  |  |  |  |  |  |  |  |  |  |  |  |  |
| *rrn23S* | ■ | ■ | ■ | ■ | ■ | ■ | ■ | ■ | ■ | ■ | ■ | ■ | ■ | ■ | ■ | ■ | ■ | ■ | ■ | ■ | ■ | ■ | ■ | ■ | ■ | ■ | ■ | ■ | ■ |
| *rrn16S* | ■ | ■ | ■ | ■ | ■ | ■ | ■ | ■ | ■ | ■ | ■ | ■ | ■ | ■ | ■ | ■ | ■ | ■ | ■ | ■ | ■ | ■ | ■ | ■ | ■ | ■ | ■ | ■ | ■ |
| *rrn5S* | ■ | ■ | ■ | ■ | ■ | ■ | ■ | ■ | ■ | ■ | ■ | ■ | ■ | ■ | ■ | ■ | ■ | ■ | ■ | ■ |  | ■ | ■ | ■ | ■ | ■ | ■ | ■ | ■ |
| **Num of tRNAs** | 28 | 33 | 27 | 35 | 31 | 31 | 27 | 27 | 27 | 27 | 31 | 37 | 30 | 31 | 25 | 28 | 30 | 31 | 36 | 28 | 30 | 27 | 25 | 32 | 36 | 37 | 33 | 37 | 37 |

**Table S2 Comparison of gene contents in microalgal mitochondrial genomes**

“■” indicate the presence of the gene on the particular mitochondrial genome, while blank indicate absence. Na: *Nannochloropsis*, Tp: *Thalassiosira*, Pt: *Phaeodactylum*, Ha: *Heterosigma*, Es: *Ectocarpus*, Fv: *Fucus*, Ot: *Ostreococcus*, Cs: *Chrysodidymus*, Cm: *Cyanidioschyzon*, Ppu: *Porphyra*, Eh: *Emiliania*, Rs: *Rhodomonas*, Sj: *Simulium*, Vc: *Volvox*, Ds: *Dunaliella*, Cr: *Chlamydomonas*, Pm: *Pedinomonas*, Pa: *Pseudendoclonium*, Pp: *Pycnococcus*, So: *Scenedesmus*, Ov: *Oltmannsiellopsis*, No: *Nephroselmis*, Mp: *Micromonas*, Cg: *Chaetosphaeridium*, Mv: *Mesostigma*, Cv: *Chara*, Ca: *Chlorokybus*, At: *Arabidopsis*.

|  | **Na** | **Tp** | **Pt** | **Ha** | **Es** | **Fv** | **Ot** | **Cs** | **Cm** | **Ppu** | **Eh** | **Rs** | **Sj** | **Vc** | **Ds** | **Cr** | **Pm** | **Pa** | **Pp** | **So** | **Ov** | **No** | **Mp** | **Cg** | **Mv** | **Cv** | **Ca** | **At** |
| --- | --- | --- | --- | --- | --- | --- | --- | --- | --- | --- | --- | --- | --- | --- | --- | --- | --- | --- | --- | --- | --- | --- | --- | --- | --- | --- | --- | --- |
| **NADH dehydrogenase** | | | | | | | | | | | | | | | | | | | | | | | | | | | | |
| *nad1* | ■ | ■ | ■ | ■ | ■ | ■ | ■ | ■ | ■ | ■ | ■ | ■ | ■ | ■ | ■ | ■ | ■ | ■ | ■ | ■ | ■ | ■ | ■ | ■ | ■ | ■ | ■ | ■ |
| *nad2* | ■ | ■ | ■ | ■ | ■ | ■ | ■ | ■ | ■ | ■ | ■ | ■ | ■ | ■ | ■ | ■ | ■ | ■ | ■ | ■ | ■ | ■ | ■ | ■ | ■ | ■ | ■ | ■ |
| *nad3* | ■ | ■ | ■ | ■ | ■ | ■ | ■ | ■ | ■ | ■ | ■ | ■ | ■ |  |  |  | ■ | ■ | ■ | ■ | ■ | ■ | ■ | ■ | ■ | ■ | ■ |  |
| *nad4* | ■ | ■ | ■ | ■ | ■ | ■ | ■ | ■ | ■ | ■ | ■ | ■ | ■ | ■ | ■ | ■ | ■ | ■ | ■ | ■ | ■ | ■ | ■ | ■ | ■ | ■ | ■ | ■ |
| *nad4L* | ■ | ■ | ■ | ■ | ■ | ■ | ■ | ■ | ■ | ■ | ■ | ■ | ■ |  |  |  | ■ | ■ | ■ | ■ | ■ | ■ | ■ | ■ | ■ | ■ | ■ | ■ |
| *nad5* | ■ | ■ | ■ | ■ | ■ | ■ | ■ | ■ | ■ | ■ | ■ | ■ | ■ | ■ | ■ | ■ | ■ | ■ | ■ | ■ | ■ | ■ | ■ | ■ | ■ | ■ | ■ | ■ |
| *nad6* | ■ | ■ | ■ | ■ | ■ | ■ | ■ | ■ | ■ | ■ | ■ | ■ | ■ | ■ | ■ | ■ | ■ | ■ | ■ | ■ | ■ | ■ | ■ | ■ | ■ | ■ | ■ | ■ |
| *nad7* | ■ | ■ | ■ | ■ | ■ | ■ | ■ | ■ |  |  |  | ■ | ■ |  |  |  |  | ■ |  |  | ■ | ■ | ■ | ■ | ■ | ■ | ■ | ■ |
| *nad8* |  |  |  |  |  |  |  |  |  |  |  | ■ |  |  |  |  |  |  |  |  |  |  |  |  |  |  |  |  |
| *nad9* | ■ | ■ | ■ | ■ | ■ | ■ | ■ | ■ |  |  |  | ■ | ■ |  |  |  |  |  |  |  | ■ | ■ | ■ | ■ | ■ | ■ | ■ | ■ |

| **LSU ribosomal proteins** | | | | | | | | | | | | | | | | | | | | | | | | | | | | |
| --- | --- | --- | --- | --- | --- | --- | --- | --- | --- | --- | --- | --- | --- | --- | --- | --- | --- | --- | --- | --- | --- | --- | --- | --- | --- | --- | --- | --- |
| *rpl2* | ■ | ■ | ■ | ■ | ■ | ■ |  | ■ |  |  |  |  |  |  |  |  |  |  |  |  |  |  |  | ■ |  | ■ | ■ | ■ |
| *rpl5* | ■ | ■ | ■ | ■ | ■ | ■ | ■ |  | ■ |  |  | ■ | ■ |  |  |  |  | ■ |  |  |  | ■ | ■ | ■ | ■ | ■ | ■ | ■ |
| *rpl6* | ■ | ■ | ■ | ■ | ■ | ■ | ■ | ■ | ■ |  |  | ■ | ■ |  |  |  |  |  |  |  |  | ■ | ■ | ■ | ■ | ■ | ■ |  |
| *rpl14* | ■ | ■ | ■ | ■ | ■ | ■ | ■ | ■ | ■ |  |  | ■ |  |  |  |  |  | ■ |  |  |  | ■ | ■ |  | ■ | ■ | ■ |  |
| *rpl16* | ■ | ■ | ■ | ■ | ■ | ■ | ■ | ■ | ■ | ■ | ■ | ■ | ■ |  |  |  |  | ■ |  |  | ■ | ■ | ■ | ■ | ■ | ■ | ■ | ■ |
| *rpl20* |  |  |  |  |  |  |  |  | ■ |  |  |  |  |  |  |  |  |  |  |  |  |  |  |  |  |  |  |  |
| **SSU ribosomal proteins** | | | | | | | | | | | | | | | | | | | | | | | | | | | | |
| *rps1* |  |  |  |  |  |  |  |  |  |  |  |  |  |  |  |  |  |  |  |  |  |  |  | ■ | ■ | ■ | ■ |  |
| *rps2* | ■ | ■ | ■ | ■ | ■ | ■ | ■ | ■ |  |  |  | ■ | ■ |  |  |  |  |  |  |  | ■ | ■ | ■ | ■ | ■ | ■ | ■ |  |
| *rps3* | ■ | ■ | ■ | ■ | ■ | ■ | ■ | ■ | ■ | ■ | ■ | ■ | ■ |  |  |  |  | ■ | ■ |  | ■ | ■ | ■ | ■ | ■ | ■ | ■ | ■ |
| *rps4* | ■ | ■ | ■ | ■ | ■ | ■ | ■ | ■ | ■ |  |  | ■ | ■ |  |  |  |  | ■ | ■ |  |  | ■ | ■ | ■ | ■ | ■ | ■ | ■ |
| *rps7* | ■ | ■ | ■ | ■ | ■ | ■ | ■ | ■ |  |  |  | ■ | ■ |  |  |  |  |  |  |  |  | ■ | ■ | ■ | ■ | ■ | ■ | ■ |
| *rps8* | ■ | ■ | ■ | ■ | ■ | ■ | ■ | ■ | ■ |  | ■ | ■ |  |  |  |  |  |  |  |  |  | ■ | ■ |  |  |  | ■ |  |
| *rps10* | ■ | ■ | ■ | ■ | ■ | ■ | ■ | ■ |  |  |  |  | ■ |  |  |  |  | ■ |  |  |  | ■ | ■ | ■ | ■ | ■ | ■ |  |
| *rps11* | ■ | ■ | ■ | ■ | ■ | ■ | ■ |  | ■ | ■ |  | ■ | ■ |  |  |  |  | ■ |  |  | ■ | ■ | ■ | ■ | ■ | ■ | ■ |  |
| *rps12* | ■ | ■ | ■ | ■ | ■ | ■ | ■ | ■ | ■ | ■ | ■ | ■ | ■ |  |  |  |  | ■ | ■ |  | ■ | ■ | ■ | ■ | ■ | ■ | ■ | ■ |
| *rps13* | ■ | ■ | ■ | ■ | ■ | ■ | ■ | ■ |  |  |  | ■ | ■ |  |  |  |  | ■ |  |  | ■ | ■ | ■ | ■ | ■ |  | ■ |  |
| *rps14* | ■ | ■ | ■ | ■ | ■ | ■ | ■ | ■ | ■ |  | ■ | ■ | ■ |  |  |  |  | ■ |  |  | ■ | ■ | ■ | ■ | ■ | ■ | ■ |  |
| *rps19* | ■ | ■ | ■ | ■ | ■ | ■ | ■ | ■ |  |  |  | ■ | ■ |  |  |  |  | ■ |  |  | ■ | ■ | ■ | ■ | ■ | ■ | ■ |  |
| **Ribosomal RNAs** |  |  |  |  |  |  |  |  |  |  |  |  |  |  |  |  |  |  |  |  |  |  |  |  |  |  |  |  |
| *rns* | ■ | ■ | ■ | ■ | ■ | ■ | ■ | ■ | ■ | ■ | ■ | ■ | ■ | ■ | ■ | ■ | ■ | ■ | ■ | ■ | ■ | ■ | ■ | ■ | ■ | ■ | ■ | ■ |
| *rnl* | ■ | ■ | ■ | ■ | ■ | ■ | ■ | ■ | ■ | ■ | ■ | ■ | ■ | ■ | ■ | ■ | ■ | ■ | ■ | ■ | ■ | ■ | ■ | ■ | ■ | ■ | ■ | ■ |
| *rrn5* |  |  |  | ■ | ■ | ■ | ■ |  | ■ |  |  |  | ■ |  |  |  |  |  |  |  | ■ | ■ |  | ■ | ■ | ■ | ■ | ■ |
| **Transfer RNAs** | 26 | 25 | 24 | 24 | 25 | 25 | 26 | 23 | 25 | 24 | 25 | 27 | 25 | 3 | 3 | 3 | 9 | 25 | 16 | 27 | 24 | 26 | 34 | 28 | 26 | 27 | 28 | 17 |

**Figure S1. Whole-organelle-genome phylogeny of *Nannochloropsis.*** All available mt and pt genomes of algae in public database to-date were included for the comparison. The trees were based on concatenated protein sequences encoded on pt (**A**) or mt (**B**). Numbers on the internal nodes represent bootstrap values (≥50%) of Maximum-Likelihood (ML), Maximum Parsimony (MP) and Neighbor-Joining (NJ).

**Figure S2. Distribution of plastid (A) and mitochondrial (B) SNPs among the seven *Nannochloropsis* strains.**

**Figure S3. The nonsynonymous (Ka) and synonymous (Ks) substitution rates of *Nannochloropsis* organelle genes.** (**A**) Plastid genes. (**B**) Mitochondrial genes. (**C**) Comparison of sequence evolution rates among plastid, mitochondrial and nuclear genes.

**Figure S4. Fine-scale structural variation of plastid IRa among the *Nannochloropsis* strains.** Insertions and deletions within the coding regions of *psbV* and *clpC* were shown. Dot: bases that are identical among the five strains. Grey background and dash: indels among the five strains. Blank box: protein-coding regions.


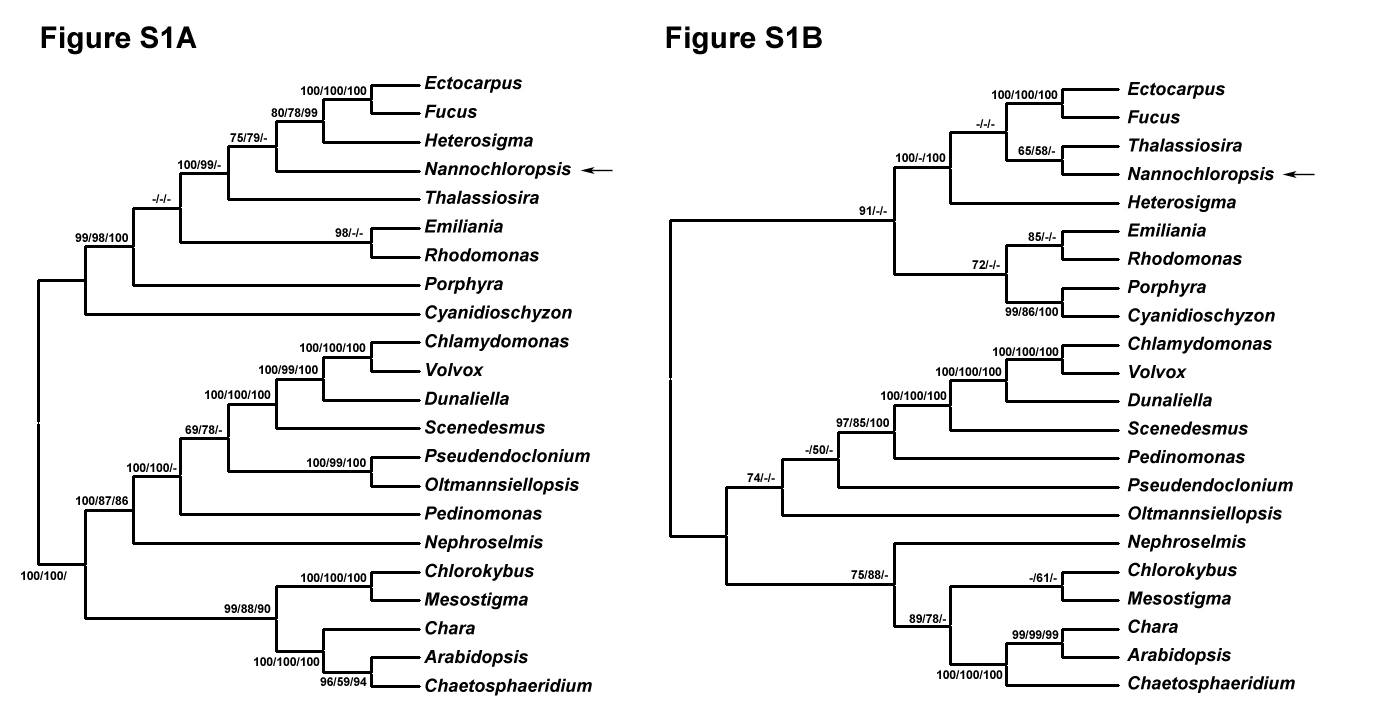

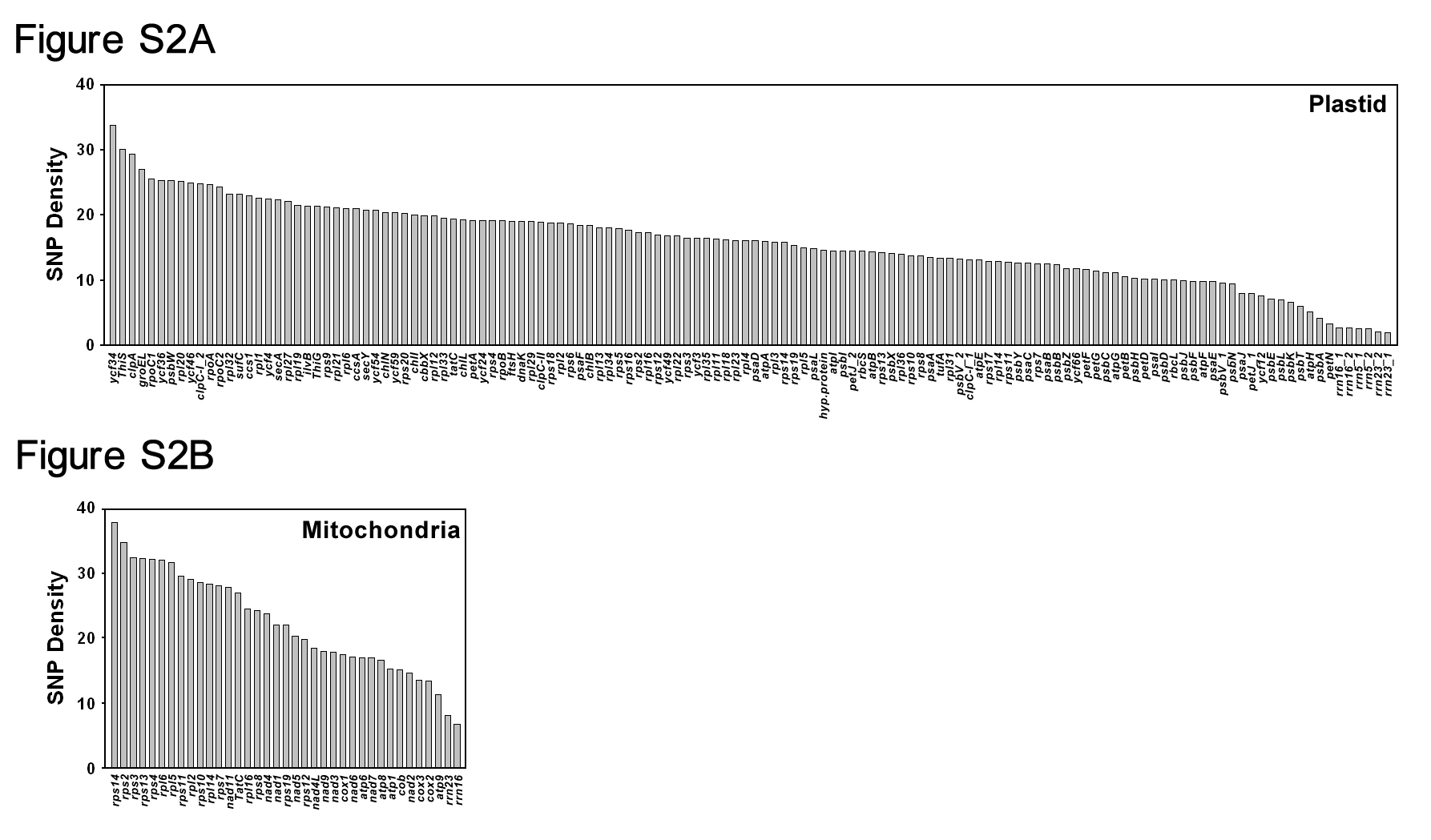

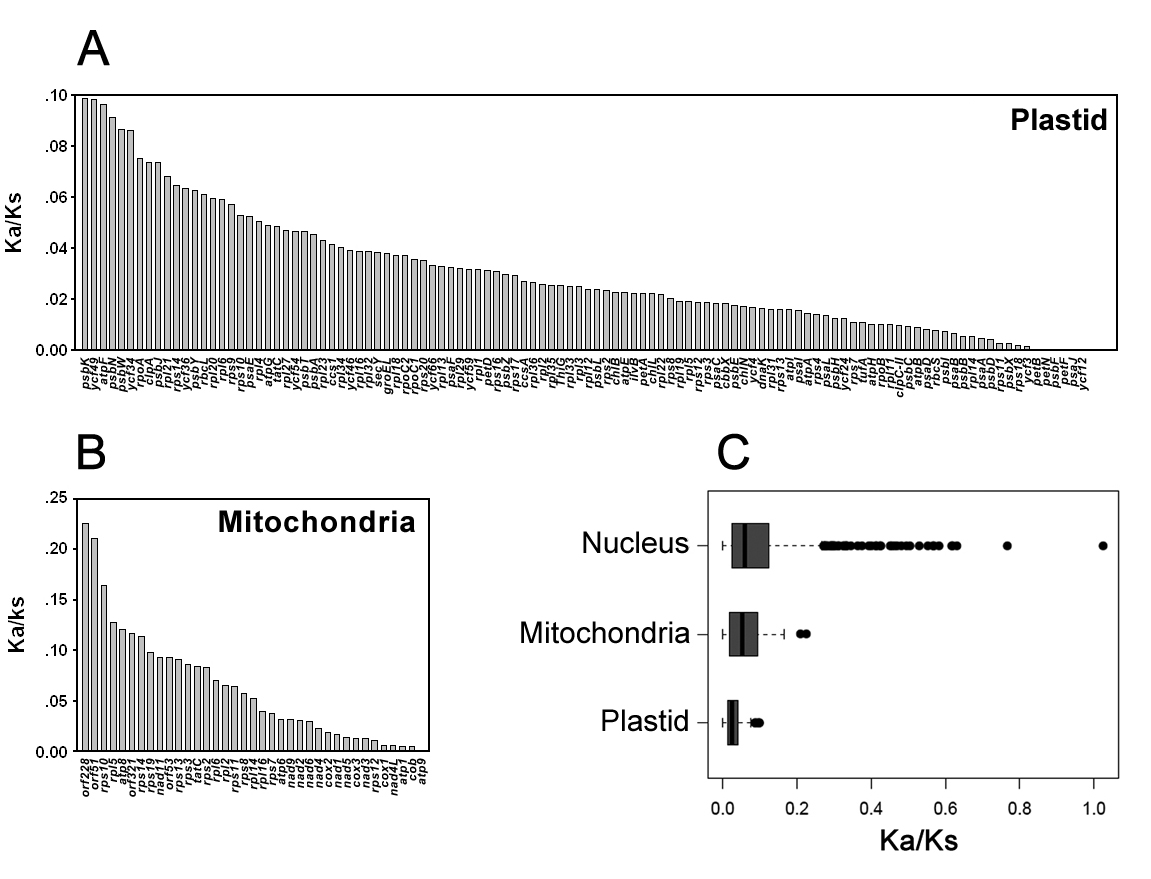

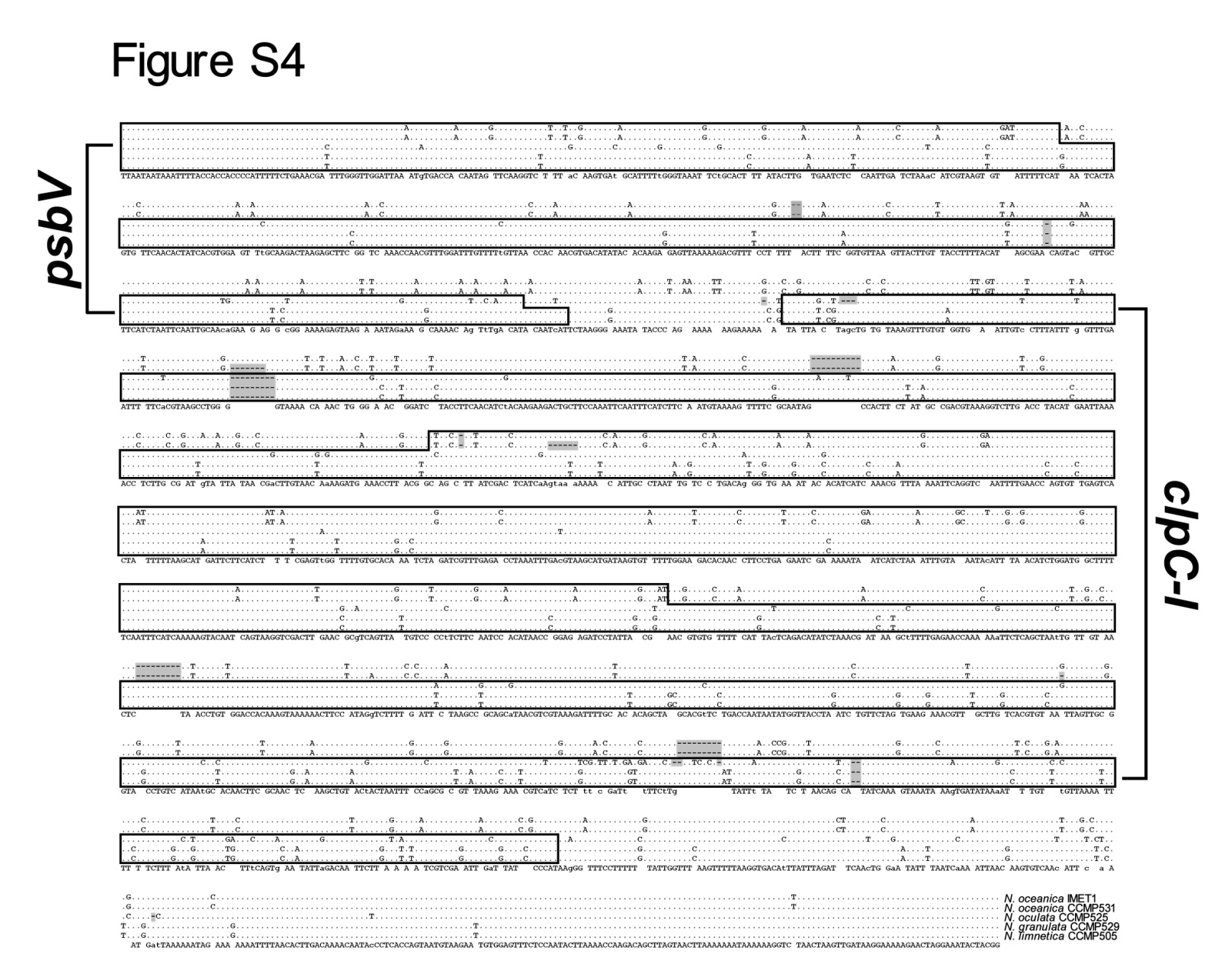

Supplement: Additional file 1 Table S1 — Comparison of gene contents in algal plastid genomes. Table S2: Comparison of gene contents in algal mitochondrial genomes. Figure S1: Whole-organelle-genome phylogeny of Nannochloropsis. All available mt and pt genomes of algae in public database to-date were included for the comparison. The trees were based on concatenated protein sequences encoded on pt (A) or mt (B). Numbers on the internal nodes represent bootstrap values (≥50%) of Maximum-Likelihood (ML), Maximum Parsimony (MP) and Neighbor-Joining (NJ). Figure S2: Distribution of plastid (A) and mitochondrial (B) SNPs among the seven Nannochloropsis strains. Figure S3: The nonsynonymous (Ka) and synonymous (Ks) substitution rates of Nannochloropsis organelle genes. (A) Plastid genes. (B) Mitochondrial genes. (C) Comparison of sequence evolution rates among plastid, mitochondrial and nuclear genes. Figure S4: Fine-scale structural variation of plastid IRa among the Nannochloropsis strains. Insertions and deletions within the coding regions of psbV and clpC were shown. Dot: bases that are identical among the five strains. Grey background and dash: indels among the five strains. Blank box: protein-coding regions. [file 1471-2164-14-534-S1.docx]
